# Development of an AIoT-Based Early Flash-Flood Warning System for Smart Rural Disaster Resilience

**DOI:** 10.3390/s26113512

**Published:** 2026-06-02

**Authors:** Visit Wiangnak, Montri Wiboonrat, Sarun Duangsuwan

**Affiliations:** 1 College of Innovation and Industrial Management, King Mongkut’s Institute of Technology Ladkrabang, Bangkok 10520, Thailand; 68186023@kmitl.ac.th; 2 KMITL Business School, King Mongkut’s Institute of Technology Ladkrabang, Bangkok 10520, Thailand; 3 Electrical Engineering, School of Engineering, King Mongkut’s Institute of Technology Ladkrabang, Prince of Chumphon Campus, Chumphon 86160, Thailand

**Keywords:** AIoT, early flash-flood warning system, rural disaster resilience, smart environment

## Abstract

**Highlights:**

**What is the main finding?**

- The proposed AIoT-based early flash-flood warning system, integrating YOLOv10 and LSTM, demonstrated high prediction accuracy and quick alert response times in real-time deployments, confirming its efficiency for early flash-flood detection and forecasting.

**What is the implication of the main finding?**

- Developing the AIoT-based early flash-flood warning system using YOLOv10 and LSTM can revolutionize rural disaster management by enabling real-time, autonomous, and community-led early warning networks, thus lowering the loss of life and property in flood-prone areas.

**Abstract:**

This paper presents the development of an AIoT-based early flash-flood warning system to enhance disaster resilience in smart rural communities. The framework integrates multi-source hydrological sensors, AI-enabled edge–cloud computing, and a mobile alert application to provide real-time monitoring and short-term flood forecasting, and includes an intelligent hybrid model combines YOLOv10 for visual water-level detection from CCTV imagery with a long short-term memory (LSTM) network for hydrological time-series prediction. The system was deployed and evaluated at two sites in Thailand: the Ban Luang station in Chiang Mai and the Chumkho station in Chumphon. The experimental results show near-perfect detection performance by YOLOv10, with precision and mAP@0.5 exceeding 0.99 across varying water-level conditions. The LSTM model achieved high forecasting accuracy, with an R^2^ of 0.987 at Ban Luang and 0.781 at Chumkho, reflecting site-specific hydrodynamic complexity. The results confirm that integrating AIoT-based visual sensing with data-driven forecasting significantly improves the reliability, responsiveness, and robustness of early flash-flood warning systems in rural environments.

## 1. Introduction

Flash floods are among the most common and destructive natural disasters in Thailand, particularly in the northern and southern provinces, which feature mountainous terrain, narrow watersheds, and rapid runoff. According to the Department of Disaster Prevention and Mitigation (DDPM), flash floods occur annually from May to November, typically triggered by short, intense rainfall events exceeding 100 mm in 1–3 h. In rural provinces, such as Chumphon, Ranong, Nakhon Si Thammarat, Chiang Mai, and Mae Hong Son, steep terrain, deforestation, and poorly designed drainage systems make local communities increasingly vulnerable to flash floods [1,2].

Over the past decade, Thailand has experienced several devastating flash floods, including the 2011 floods, which affected more than 800,000 households and caused economic losses exceeding THB 30 billion. In 2021, a flash flood inundated farmland, disrupted transportation networks, and damaged irrigation systems. These recurring events highlight systemic weaknesses in hydrological monitoring coverage, real-time data transmission, and community-level alert dissemination, often resulting in delayed evacuations and inadequate disaster preparedness [3,4].

Despite advances in meteorological forecasting, most traditional systems still rely on centralized hydrological models that cannot fully capture local rainfall–runoff dynamics in small rural watersheds. Rural areas remain underserved because rugged terrain and low population density hinder the installation of continuous observation stations and reliable communication networks. Therefore, there is an urgent need for decentralized, low-cost, and intelligent early-warning systems that can process localized environmental data and deliver timely alerts directly to at-risk communities [4].

Recent advances in the Internet of Things (IoT) and Artificial Intelligence (AI) have created transformative opportunities for real-time environmental sensing and predictive analytics [5,6]. IoT-based monitoring systems continuously collect data on rainfall, temperature, humidity, and water levels, while AI models analyze temporal patterns to forecast the flood risk. The integration of these technologies, known as AIoT, forms an interconnected framework for autonomous, data-driven early-warning systems for applications in agriculture [7] and disaster prevention [8].

Although previous studies have demonstrated the feasibility of IoT-based flood monitoring [9,10,11], only a limited number have implemented AIoT-driven systems that integrate intelligent edge computing across diverse hydrological conditions [8,10,11]. Most of the existing systems rely solely on cloud-based analysis and static thresholds, which are prone to latency and communication interruptions, especially in remote rural regions. Moreover, there is no comprehensive framework that unifies IoT sensing, AI-edge computing, and automated multi-platform alerting for localized flash-flood warnings in rural communities.

To address the identified challenges, this study proposes developing an AIoT-based early flash-flood warning system (EFFWS) that integrates YOLOv10 and LSTM algorithms for intelligent flood detection and forecasting.

The key research contributions of this work are as follows.

- The architecture incorporates an ultrasonic water-level sensor, CCTV cameras, a rain gauge, an environmental sensor, and a small AI edge server connected via a 5G network, ensuring high-speed, low-latency data transmission for alerting flood potential. Although LPWAN technologies such as LoRaWAN provide energy-efficient, long-range communication for low-data-rate environmental sensing, the proposed framework also requires real-time CCTV video transmission, AI edge synchronization, cloud visualization, and low-latency mobile alert dissemination. Therefore, 5G communication is selected in this work for its ability to support high-bandwidth, low-latency AIoT operations. Nevertheless, the proposed framework remains communication-layer-flexible and can be adapted to 3G, 4G, or LPWAN infrastructures, depending on deployment requirements and rural communication constraints.
- The data pipeline, built with Node-RED, InfluxDB, and Grafana, enables seamless data flow, storage, visualization, and system automation, while messaging integration delivers instant alerts to communities and local authorities.
- The AIoT model uses YOLOv10 for real-time visual flood detection from CCTV cameras. By contrast, the LSTM model predicts water-level trends.
- The experimental setup was tested at two locations: Ban Luang district in Chiang Mai and Chumkho district in Chumphon province.
- The key performance results present YOLOv10’s metrics as precision, recall, and mAP. By contrast, the LSTM metrics are reported as RMSE, MAE, and R^2^.

The rest of this paper is organized as follows: Section 2 reviews the related works; Section 3 presents a schematic of the early flash-flood warning system; Section 4 explains the AIoT model, including YOLOv10 and the LSTM model; Section 5 describes the experimental setup; Section 6 presents the results and Section 7 discusses them; finally, Section 8 presents the conclusion.

## 2. Related Works

Recent research on flood monitoring and early-warning systems has advanced through the integration of IoT, machine learning (ML), and edge computing. This section reviews the relevant literature across the following three domains: IoT-based hydrological monitoring, AI-driven flood forecasting, and computer vision and hybrid AI approaches for real-time flood detection.

### 2.1. IoT-Based Flood Monitoring Systems

Recent advances in IoT-based flood monitoring have significantly enhanced real-time sensing and community-centered disaster management. Pillay et al. [12] emphasized the design of affordable IoT flood stations equipped with ultrasonic sensors to provide an accessible early warning system for developing regions, although these stations lack predictive analytics. Similarly, Zakaria et al. [13] introduced a LoRaWAN-based IoT monitoring network that proved reliable for long-range communication but was limited to data collection without AI-driven analysis. To improve sustainability, Ragnoli et al. [14] proposed an autonomous, solar-powered LoRa node system optimized for low energy consumption; however, it did not incorporate multi-sensor fusion or intelligent decision-making. Recent studies have increasingly explored hybrid integration between LPWAN and next-generation cellular networks for environmental monitoring and smart-city applications. Das et al. [15] discussed the role of scalable AIoT communications infrastructure in supporting sustainable smart-city transformation through heterogeneous wireless technologies. Giacobbe et al. [16] demonstrated decentralized LoRaWAN-based wildfire monitoring for long-range environmental sensing in remote forest areas. Shayea et al. [17] further highlighted the emerging integration of 5G/6G, IoT, and LEO communication systems for resilient large-scale monitoring applications. These studies indicate that hybrid communication architectures combining LPWAN and cellular infrastructure are becoming increasingly important for scalable rural AIoT deployments. Anbarasan et al. [18] investigated the use of IoT and deep learning for large-scale flood analysis using big data; however, their approach’s reliance on cloud infrastructure limited its real-time use in rural areas. More recently, Choosumrong et al. [19] developed an IoT–GIS flood-mapping platform for Thailand’s lowland agricultural zones, which enhances visualization but lacks autonomous edge processing and visual detection capabilities. Complementary reviews by Bukhari et al. [20] and Bakhtiari et al. [21] highlight that, while IoT systems offer robust sensing and communication capabilities, they still lack decentralized AI-based intelligence and localized alert distribution.

### 2.2. AI and ML Techniques for Flood Prediction

AI and ML techniques have revolutionized flood forecasting by enabling pattern detection and adaptive learning from hydrological data. Mosavi et al. [22] provided a comprehensive review of over 180 studies applying ML algorithms, including artificial neural networks (ANNs), support vector machines (SVMs), adaptive neuro-fuzzy inference systems (ANFISs), and wavelet neural networks (WNNs), to flood prediction. They concluded that hybrid models outperform traditional statistical methods but remain limited in real-time, sensor-based deployment. Building on this, Al-Rawas et al. [23] explored emerging AI technologies for flash-flood forecasting. They highlighted challenges related to data diversity, feature extraction, and implementation, and emphasized the need for IoT-integrated AI systems to improve regional scalability.

Tabbussum and Dar [24] compared neural networks, fuzzy systems, and ANFIS, and demonstrated that hybrid neuro-fuzzy frameworks effectively capture complex, non-linear rainfall–runoff relationships. However, they require significant computational resources and cloud resources. Kumar et al. [25] summarized the advances in deep learning, including convolutional neural networks (CNNs), recurrent neural networks (RNNs), and LSTM, which demonstrate superior accuracy but have weaknesses in interpretability and decentralized processing. Field implementations, such as those by Prakash et al. [26] with FLOODWALL and Faudzi et al. [27], have confirmed that LSTM-based IoT systems can reliably forecast water-level dynamics; however, both lacked vision-based situational awareness. Kim et al. [28] employed deep neural networks (DNNs) and ensemble models, including random forests (RFs) and XGBoost, for flood-risk classification. Ahmed et al. [29] employed a DeepLabv3 CNN on IoT data for flood-extent mapping, though both remained cloud-dependent.

The most advanced hybridization, proposed by Arante et al. [30], combined LSTM, fuzzy logic, and genetic algorithms into a secure IoT architecture, achieving 92.9% prediction accuracy but still requiring centralized computation. Overall, these studies [22,23,24,25,26,27,28,29,30] show that, while AI and ML techniques significantly improve predictive accuracy, the current models lack real-time, edge-AI independence and community-level responsiveness.

### 2.3. Computer-Vision and Hybrid AI Approaches

Rapid advances in computer vision (CV) and hybrid AI have significantly enhanced real-time flood detection and situational awareness. Arshad et al. [31] provided one of the earliest comprehensive reviews combining IoT and CV for flood monitoring, summarizing over 90 studies and concluding that image-based systems offer low-cost, scalable solutions when integrated with sensor networks. However, challenges in data reliability and edge implementation persist. Borwarnginn et al. [32] developed a CNN-based water-level estimation model using CCTV footage in Thailand, achieving 93% accuracy with single-camera setups and 83% accuracy with multiple-camera setups. They showed that vision-only detection could replace traditional sensors in resource-limited areas.

Jan et al. [33] introduced an edge-AI framework that uses RGB-D cameras with stereoscopic capabilities to measure water levels in open areas, enabling local processing for rapid flood alerts. Their method reduced bandwidth usage but remained vulnerable to environmental noise. Zhao et al. [34] combined CNN and LSTM networks for nationwide flash-flood prediction, achieving a hit rate of 0.84 and a false-alarm rate of 0.09. This demonstrated the advantages of hybrid temporal–spatial modeling but still relied on centralized training servers.

Recent studies have focused on deep learning for visual flood classification [35,36,37,38]. Kwon and Lee [35] proposed a U-Net segmentation model for CCTV-based water-level recognition in agricultural reservoirs, achieving high segmentation accuracy but requiring a large, well-annotated dataset. Wan et al. [36] trained YOLOv8 on 6300 flood-vehicle images, achieving 95% accuracy; however, its urban focus limits applicability in rural landscapes. Similarly, Teoh et al. [37] used generative AI to augment flood datasets, improving YOLOv8 robustness to 96.7% accuracy, though this relied on cloud-dependent computation. Recently, Ramos et al. [38] combined IoT sensors with YOLOv8–SVM hybrid models for flood and debris monitoring, achieving 100% hydrological and 83% visual accuracy, though the performance levels dropped under low-light conditions.

Collectively, these studies [31,32,33,34,35,36,37,38] confirm that CV and hybrid AI systems have improved flood prediction accuracy and have enabled automated flood prediction. However, they still face challenges, including urban bias, dependence on cloud computing, and limited effectiveness in rural areas. The present study builds on these findings by integrating YOLOv10 for real-time visual detection [39] and LSTM [26,27,30,40] for hydrological forecasting into a unified AIoT edge framework, providing autonomous, low-latency flood warnings tailored explicitly for rural communities.

### 2.4. Research Gap and Distinct Contribution

The reviewed literature [12,13,14,15,16,17,18,19,20,21,22,23,24,25,26,27,28,29,30,31,32,33,34,35,36,37,38] indicates that existing IoT-based systems offer valuable sensing capabilities but lack intelligent event prediction [24,32,37,38]. By contrast, AI-driven models often operate offline in centralized cloud environments [27,28,29,30,32]. Similarly, most computer vision approaches focus on urban areas [27,36,37,38,39,40], rather than on community-based early warning systems. Therefore, there is a significant gap in developing a hybrid AIoT architecture for flood monitoring that accomplishes the following:

- Combines IoT-based hydrological sensing with real-time computer vision;
- Performs on-device, edge-level AI inference with lightweight models, such as YOLOv10 and LSTM;
- Enables automatic alert distribution via notifications.

This paper addresses these limitations by designing an AIoT-based early flash-flood warning system that uses YOLOv10 for CCTV-based flood detection and an LSTM model for hydrological time-series forecasting. It is implemented on a small edge server with integrated data-flow management using Node-RED, InfluxDB, and Grafana. This framework offers a new hybrid intelligence for decentralized, real-time flood prediction and community-responsive warning dissemination in smart rural areas. Table 1 compares the existing flood early warning systems with the proposed AIoT framework.

## 3. Early Flash-Flood Warning System

### 3.1. System Architecture

The early flash-flood warning system is designed for integrating real-time environmental sensing, computer vision, and predictive analytics. The objective is to deliver autonomous, low-latency alerts to rural communities under imminent flash-flood threats.

**Figure 1:**
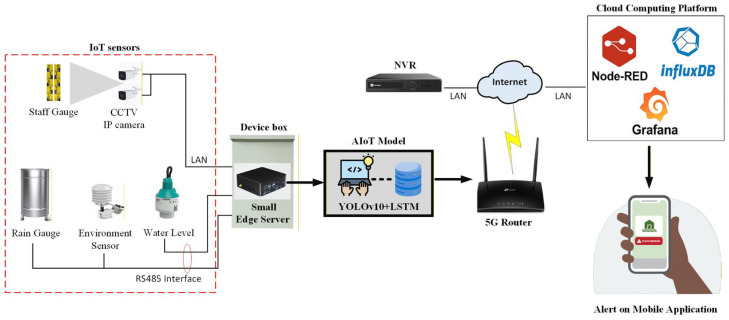
System architecture of an early flash-flood warning system.

Figure 1 illustrates the architecture of an early flash-flood warning system that integrates multi-sensor IoT monitoring, high-resolution CCTV surveillance, an AIoT model, cloud computing, and mobile alert dissemination. The system is designed to operate in rural environments where reliability, low latency, and autonomous operation are essential. The system architecture consists of the following four primary components:

1. IoT sensing layer:
  The system begins with a suite of hydro-meteorological IoT sensors installed at each monitoring station:
  - A staff gauge provides a visual reference of the water height for cross-validation with CCTV images.
  - CCTV IP cameras continuously capture video streams of river channels. The YOLOv10 model subsequently analyzes these images to detect rapid increases in water level, flow, or changes in turbidity.
  - A rain gauge measures the real-time rainfall accumulation with high temporal resolution.
  - An environmental sensor monitors the ambient temperature, light, and humidity to support flood prediction models and sensor calibration.
  - An ultrasonic water-level sensor measures the water level across short intervals using non-contact sonar. It communicates via an industrial RS485 interface, ensuring stable operation in harsh outdoor environments.
  - The small edge server provides control of the CCTV IP cameras and IoT sensors inside the device box.
2. Communication and networking layer:
  A 5G router serves as the core of the communication layer, enabling high-throughput, low-latency connectivity among the monitoring site, AI edge server, and cloud computing platform. Unlike conventional LPWAN systems designed primarily for low-data-rate sensing, the proposed framework additionally supports real-time CCTV video streaming, AIoT synchronization, cloud visualization, and rapid mobile alert dissemination. Therefore, 5G communication is selected to ensure reliable low-latency operation under practical flood-monitoring conditions.
  - A 5G cellular network for rapid upstream video and sensor transmission.
  - Low latency for real-time inference.
  - Reliable connectivity for rural areas.
  - A network video recorder (NVR) is connected via the local area network (LAN) cable to store the CCTV video streams locally. This ensures continuous video logging even during temporary network interruptions.
3. Small edge server layer:
  The central intelligence of the system resides in the AIoT model, which runs the following two models:
  - The YOLOv10 model analyzes CCTV frames in real time to detect water-level anomalies, water flow, fast-moving currents, and debris accumulation.
  - The LSTM flood forecasting model performs time-series prediction using rainfall, temperature, and water-level data from IoT sensors, estimating flood likelihood minutes to an hour ahead.
4. Cloud computing platform and alerting user interface:
  The system triggers automated alerts delivered through the following:
  - Node-Red, InfluxDB, and Grafana dashboard application.
  - Line messaging notification.

### 3.2. Hardware Specification

**Figure 2:**
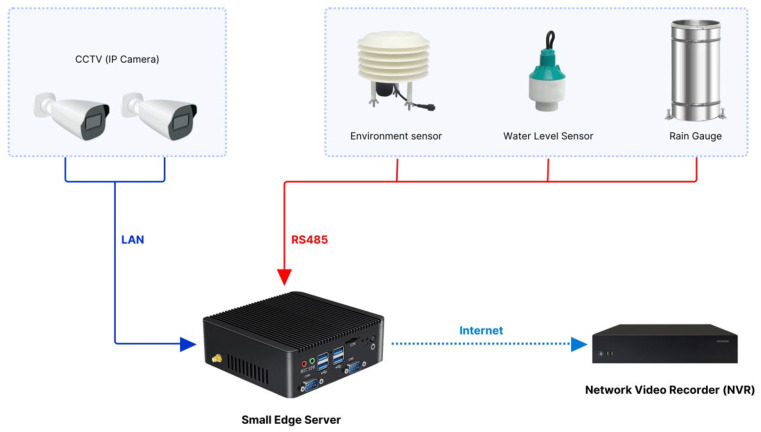
Integration of IoT sensors with the small edge server used in the early flash-flood warning system.

Figure 2 illustrates the integration of multiple IoT sensing devices with a small edge server that processes, stores, visualizes, and enables automated alerting.

1. CCTV IP cameras:

High-definition IP CCTV cameras serve as the primary imaging devices for detecting rapid changes in flood warning and water levels. The specifications are as follows: video resolution supports 1080P and 720P, a DC 12-volt power supply is supported, and a micro–SD card supports local storage. The operating temperature is 0–50 °C. The NVR is connected via an LAN cable to store CCTV video streams.

2. Rain gauge:

A tipping-bucket rain gauge is installed to acquire rainfall accumulation with high temporal accuracy. The specifications feature a support 12 VDC power supply and a rain collector capacity of 200 mm, which measure rainfall intensity in the range of 0–3 mm/min. The communication port is an RS485 Modbus RTU port. The operating temperature is 0–50 °C, and the relative humidity is <95% at 40 °C. Figure 2 shows the sample rain gauge.

3. Environmental sensor:

An environmental sensor monitors the ambient temperature and relative humidity. This contextual climate data helps calibrate the hydrological prediction models and ensures stable sensor performance with the capability of measuring solar light intensity in the range of 0–200,000 lux. The communication port uses an RS485 Modbus RTU protocol. The power supply supports a DC voltage range of 10 to 30 V, temperature is measured from 0 to 50 °C, and relative humidity is measured from 0 to 99%.

4. Ultrasonic water level:

An ultrasonic water level sensor measures instantaneous changes in the water stage using non-contact ultrasonic ranging. This sensor provides high-accuracy time-series data that feeds directly into the LSTM-based flood-forecasting algorithm. The Siemens SITRANS LR100 (Munich, Germany) ultrasonic water level brand is used. The product is waterproof, with an IP65 protection rating. The water-level range is 0–8 m, the power supply supports 12–35 V DC, and the communication port uses an RS485 Modbus RTU protocol.

5. Small edge server:

The small edge server functions as the central computational unit that bridges field IoT sensing, edge-level machine intelligence, and cloud-based analytics.

The hardware specification for a small edge server is as follows: the CPU has four cores and four threads, a clock speed of 2 GHz, 4 MB of cache, and an integrated graphics processor. The storage is 128 GB of solid-state storage (SSD), and the network interface is 10/100/1000 base-T Ethernet. It includes five USB 3.0 interface ports and three USB 2.0 ports, one HDMI port, three RS232 ports, and three RS485 ports. The operating system is Linux, and the communication network supports 4G and 5G networking.

### 3.3. Data Flow and Processing Pipeline

**Figure 3:**
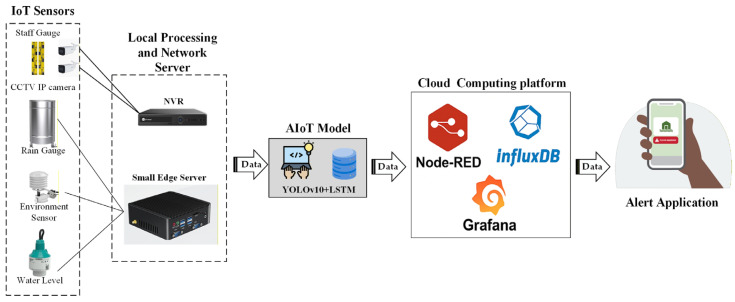
Data flow and processing pipeline.

The data flow and processing pipeline of the early flash-flood warning system is designed to ensure efficient acquisition, transmission, analysis, and dissemination of hydrological information in real time. The pipeline integrates multi-sensor IoT data, CCTV imagery, edge computing, and cloud-native microservices to support autonomous decision-making and low-latency community alerts. The entire process comprises five sequential stages, as illustrated in Figure 3.

1. IoT sensor data acquisition layer:

The IoT sensing layer underpins the early flash-flood warning system by continuously monitoring hydrological and environmental conditions at the river site. This layer integrates heterogeneous devices, including a staff gauge, CCTV IP cameras, a rain gauge, an environmental sensor, and an ultrasonic water-level sensor. The CCTV cameras capture real-time images of the river and gauge markings, enabling visual extraction of water levels, while the water-level sensor provides precise depth measurements. The rain gauge records precipitation intensity, and the environmental sensor measures temperature, humidity, and pressure, all of which influence short-term flood behavior. Sensor data is transmitted via RS485 and LAN connections to the local processing and networking server. By combining visual and numerical observations, the IoT sensing layer provides rich, multimodal information for downstream AI models and cloud analytics, forming a robust basis for accurate flood detection and predictive forecasting.

2. Local processing and network server layer:

The local processing and networking server serves as the intermediary hub between field IoT devices and the AIoT model. This subsystem comprises two main components, namely the NVR and the small edge server. The NVR receives continuous streams from CCTV cameras, extracts frames for inference, and stores footage for verification or retraining. The small edge server collects raw sensor data via RS485 and LAN interfaces, synchronizes timestamps, filters out noise, and preprocesses all inputs before AIoT analysis. This edge device ensures reliable data handling even under unstable network conditions, and reduces the computational burden on cloud systems by handling local preprocessing tasks. Additionally, by maintaining real-time communication channels, the local server ensures that data streams from multiple sensors are synchronized and correctly formatted for input to the AIoT model, thereby enabling low-latency, high-accuracy flash-flood monitoring.

3. AIoT model:

The AIoT model integrates YOLOv10 for visual flood detection and LSTM for hydrological time-series forecasting, forming a hybrid intelligence engine that enhances the accuracy and responsiveness of the early warning system. YOLOv10 processes CCTV images to identify the staff gauge and to detect water-level markings by leveraging multi-scale feature extraction across its backbone, neck, and detection head modules. This yields a visual estimate of the water level and flood classes (low, mid, and high). Complementing this, the LSTM model analyzes sequences of numerical sensor data, including rainfall, temperature, humidity, light, and water depth, to learn temporal patterns and to predict future water levels over a defined forecast horizon. The outputs of YOLOv10 and LSTM can be fused to yield a more reliable flood assessment that captures both instantaneous visual changes and longer-term hydrological trends. This combined AIoT model supports proactive alerts, enabling the system to detect sudden increases in water level and to forecast potentially dangerous conditions in advance.

4. Cloud computing platform:

The cloud computing platform manages long-term data storage, data visualization, system orchestration, and automated alerts. It is built on three core modules, namely Node-RED, InfluxDB, and Grafana. Node-RED orchestrates the flow of processed data from the AIoT model to cloud services and implements rules-based logic to evaluate water-level thresholds, prediction outcomes, and flood-risk conditions. InfluxDB is a time-series database that stores multimodal hydrological data and AIoT outputs, enabling real-time queries and historical analytics. Grafana provides intuitive dashboards that visualize river conditions, rainfall patterns, LSTM predictions, and YOLOv10 detection results, helping officials and researchers to monitor system performance and community risk levels. These cloud services enable scalable data management, remote access, and integration with emergency communication systems. The platform ensures that real-time alerts are generated promptly and that historical data remain available for model improvement, community planning, and disaster-resilience studies.

5. Alert application:

The alert application is the final stage of the EFFWS process, delivering actionable, real-time notifications to community members, local authorities, and disaster response teams. Based on outputs from the YOLOv10 and LSTM models, including predicted water levels, visual flood classifications, and threshold-exceedance conditions, Node-RED triggers automated warnings sent directly to mobile devices. These warnings include flood alerts, advisory messages, and, when applicable, visual data. The mobile interface is designed for simplicity and rapid comprehension, enabling timely responses during emergencies. By delivering alerts via line notification, the system provides broad coverage in rural communities, where rapid dissemination of flood warnings is critical to minimizing loss and enhancing preparedness. The alert application thus serves as the operational endpoint of the AIoT-based early flash-flood warning system, translating advanced analytics and predictive models into practical, life-saving information for end users. Open-source platforms, including Node-RED, InfluxDB, Grafana, and LINE messaging, are intentionally selected to improve affordability, scalability, flexibility, and ease of deployment in rural environments. These commodity software tools reduce implementation cost, simplify maintenance, support rapid system customization, and improve reproducibility for community-level AIoT disaster-resilience applications.

## 4. AIoT Model

This section presents the AIoT model for an early flash-flood warning system and short-term hydrological forecasting. The system integrates two complementary AIoT models: YOLOv10, which processes CCTV imagery to detect visible signs of flooding, and LSTM networks for time-series predictions of water levels, rainfall, and environmental sensor data. A modified hybrid architecture that combines both models is then proposed to enhance accuracy, robustness, and responsiveness under rural ecological constraints.

### 4.1. YOLOv10

YOLOv10 [41] is the primary computer vision model for detecting flood-related objects and water-level changes in CCTV streams. As the latest evolution in the YOLO family, YOLOv10 integrates anchor-free detection, structural reparameterization, and dynamic label assignment, delivering higher accuracy and lower latency than earlier versions [37,38]. YOLOv10 introduces speed–accuracy efficiency optimization (SAEO) and progressive model compression, making it the most efficient version of YOLO for edge computing. It delivers superior real-time performance, surpassing YOLOv9 while maintaining comparable or higher accuracy. YOLOv10 is highly resilient to environmental noise, low visibility, nighttime CCTV, and rainfall-induced occlusion, conditions common in flash-flood environments. YOLOv10 is therefore ideal for deployment on industrial mini-PCs, Jetson modules, and on-site flood-monitoring devices where power and bandwidth are limited. Table 2 presents the comparative performance of the YOLO variants, including YOLOv5, YOLOv7, YOLOv8, YOLOv9, and YOLOv10.

**Figure 4:**
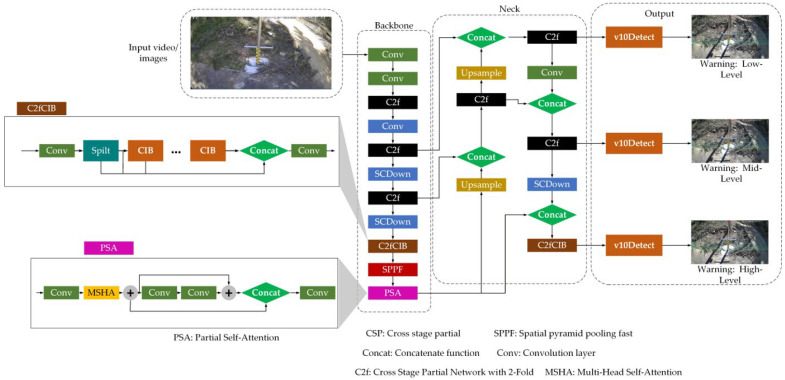
The YOLOv10 model.

Figure 4 presents the complete architecture of the YOLOv10 model used for real-time flood detection from CCTV video streams. The model consists of the following four main components: (1) an input state, (2) a backbone for hierarchical feature extraction, (3) a neck for multi-scale feature aggregation, and (4) a v10Detect output head for object detection at multiple staff gauge levels. Several improved modules are integrated to enhance robustness in outdoor environments typical of flood monitoring sites.

1. Input state:

The model takes video frames or images from CCTV cameras as input. These inputs typically capture the dynamic water level at staff gauges.

2. Backbone state:

The backbone performs feature extraction through multiple convolutional and cross-stage partial (CSP) blocks, learning low-, mid-, and high-level features.

The key modules include the following:

- Conv is the standard convolutional layer for initial feature encoding.
- C2fCIB is a cross-stage partial module with convolutional information blocks that improve gradient flow and reduce computational redundancy.
- SCDown is a spatial-channel decoupled down-sampling method that preserves both spatial and channel information when reducing resolution.
- SPPF pools features across different receptive field sizes to capture multi-scale contextual information, which is crucial for identifying turbulent water movement and overflowing rivers.
- PSA applies multi-head attention to enhance feature representation, especially under occlusion, low-light, or heavy-rain conditions.

3. Neck state:

The neck fuses feature maps from different scales via up-sampling, down-sampling, and concatenation to enhance the network’s ability to detect targets of varying sizes.

The key operations include the following:

- Concatenation merges feature maps from different depths to preserve detail across multiple scales.
- Up-sampling ensures consistent resolution alignment between the feature maps.
- C2f blocks improve feature fusion and stabilize multi-layer communication.

Note that this Neck structure forms a bidirectional, multi-scale representation, enabling the model to detect both fine-scale waterline changes and broader flood-spread patterns.

4. Output head:

The v10Detect head produces multi-scale detection outputs corresponding to the visual water-level conditions observed at the staff gauge. In this work, the YOLOv10 framework performs binary visual classification between low- and high-water conditions. The intermediate medium-level warning state is subsequently generated during the fusion and threshold evaluation stage by integrating sensor measurements, LSTM forecasting outputs, and YOLOv10 visual detection results, according to predefined warning thresholds.

### 4.2. Hybrid YOLOv10 and LSTM Model

An LSTM network is a recurrent neural network (RNN) designed to capture non-linear temporal dependencies in sequential data [24,32]. In flash-flood forecasting, hydrological variables, such as rainfall, water levels, humidity, and temperature, evolve, with short-term spikes followed by delayed water-level responses. Standard RNNs struggle with long-range temporal dependencies; therefore, LSTMs introduce gate mechanisms and an internal memory state to preserve critical information across multiple time steps.

**Figure 5:**
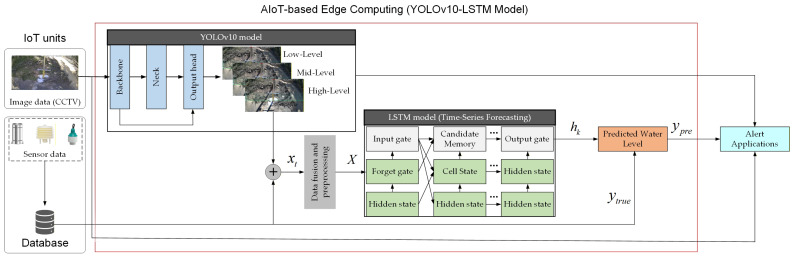
The architecture of the AIoT-based edge computing system that combines YOLOv10 and LSTM for an early flash-flood warning system.

Figure 5 illustrates the architecture of the proposed AIoT-based edge computing system, which combines YOLOv10 with LSTM to enable early flash-flood warnings. The structure consists of the following three main sections: (1) the YOLOv10 model, (2) the LSTM model, and (3) output predictions that represent the performance matrices.

The system operates as a sequential pipeline in which visual features from the YOLOv10 model are fused with sensor data to drive the LSTM forecasting engine. For every detected staff gauge at the time step t, the model generates a composite output vector $O_{t}$ containing both the spatial bounding line coordinates and the class probabilities as follows:

(1) $$O_{t}=\left[bx_{t},by_{t},bw_{t},bh_{t},P_{low},P_{mid},P_{high}\right]$$

where $\left(bx_{t},by_{t}\right)$ are the center coordinates of the detected water region, $\left(bw_{t},bh_{t}\right)$ are the width and height of the bounding lines, respectively, and $\left(P_{low},P_{mid},P_{high}\right)$ denote the confidence probabilities corresponding to the three danger levels, subject to the constraint $\sum P_{c}=1$.

From the detected bounding lines, we extract the visual water level height $H_{vis}\left(t\right)$, which is derived using the argument of the maximum function, and is given as follows:

(2) $$H_{vis}\left(t\right)=\underset{c\in \left\{low,high\right\}}{\arg\max}\left[P_{c}\left(t\right)\right]$$

According to the data fusion and preprocessing block in Figure 5, the system aggregates the visual data with physical sensor data. We define the feature vector $x_{t}$ at the time step t as follows:

(3) $$x_{t}={\left[H_{vis}\left(t\right),H_{sens}\left(t\right),R_{t},E_{t}\right]}^{T}$$

where $H_{sens}\left(t\right)$ is the water level from the ultrasonic sensor, $R_{t}$ is the data from the rain gauge sensor, and $E_{t}$ is the data from the environment sensor, such as temperature, humidity, and solar light intensity.

The input LSTM model processes a sequence of these vectors over a look-back window of size k, as follows:

(4) $$X=\left[x_{t-k},x_{t-k+1},x_{t-k+2},\ldots ,x_{t}\right]$$

Since the hydrological sensor data were recorded at 1-h intervals, we set k=24, corresponding to the previous 24 h of water level, rainfall, temperature, humidity, and light-intensity observations.

The forecasting objective is to predict the water level at a future horizon (H). In this study, the forecast horizon is set to H=1, which corresponds to the 1-h-ahead water-level prediction, since the dataset was recorded at 1-h intervals.

For each input $x_{t}$, the LSTM cell computes the following gate operations:

- Forget gate ($f_{t}$):

For flash-flood monitoring, the forget gate plays a critical role in handling the non-stationarity of the hydrological data. It allows the model to discard historical rainfall data that has already contributed to runoff and is no longer relevant to the current water level, thereby preventing the accumulation of obsolete temporal dependencies as follows:

(5) $$f_{t}=\sigma \left(W_{f}\cdot \left[h_{t-1},x_{t}\right]+b_{f}\right)$$

where σ denotes the sigmoid activation function [32], $W_{f}$ is the weight function, $h_{t-1}$ is the previous hidden state, and $b_{f}$ is the bias function.

- Input gate ($i_{t}$) and candidate memory (${\tilde{C}}_{t}$):

The input gate and cell state operate in tandem to integrate new environmental data into the system. The cell state utilizes a hyperbolic tangent function (tanh) to model the magnitude and direction of state changes (e.g., rising or receding water levels). At the same time, the sigmoid-based input gate regulates the flow of this information, ensuring that only significant hydrological events effectively update the long-term cell state ($c_{t}$). This can be expressed as follows:

(6) $$i_{t}=\sigma \left[W_{i}\cdot \left[h_{t-1},x_{t}\right]\right]+b_{i}$$

(7) $${\tilde{C}}_{t}=\tanh\left(W_{c}\cdot \left[h_{t-1},x_{t}\right]+b_{c}\right)$$

(8) $$c_{t}=f_{t}⊙c_{t-1}+i_{t}⊙{\tilde{C}}_{t}$$

where ⊙ denotes element-wise multiplication.

- Output gate ($O_{t}$):

While the input and forget gates control the candidate memory (${\tilde{C}}_{t}$), the output gate controls the external output. It decides what information is relevant to pass to the next time step and, crucially, what information is used to make the actual water level prediction. This can be expressed as follows:

(9) $$O_{t}=\sigma \left(W_{o}\cdot \left[h_{t-1},x_{t}\right]+b_{o}\right)$$

(10) $$h_{t}=O_{t}⊙\tanh\left(c_{t}\right)$$

- Prediction output ($y_{pre}$):

The final stage of the forecasting module involves mapping the high-dimensional hidden state ($h_{t}$) to a scalar hydrological value. This is achieved through a fully connected layer, defined as follows:

(11) $$y_{pre+H}=W_{out}\cdot h_{t}+b_{out}$$

where $W_{out}$ and $b_{out}$ represent the regression weights and bias, respectively. Unlike the internal gates that utilize non-linear activations (σ,tanh), the output layer employs a linear activation function to regress the specific water level value. This predicted value $y_{pre}$ is subsequently fed into the threshold logic unit to determine the appropriate alert status.

## 5. Experimental Setup

This section presents the experimental setup, including visual flood detection using YOLOv10, hydrological time-series forecasting using LSTM, and the overall system performance in rural environments.

### 5.1. Site Implementation in Rural Communities

**Figure 6:**
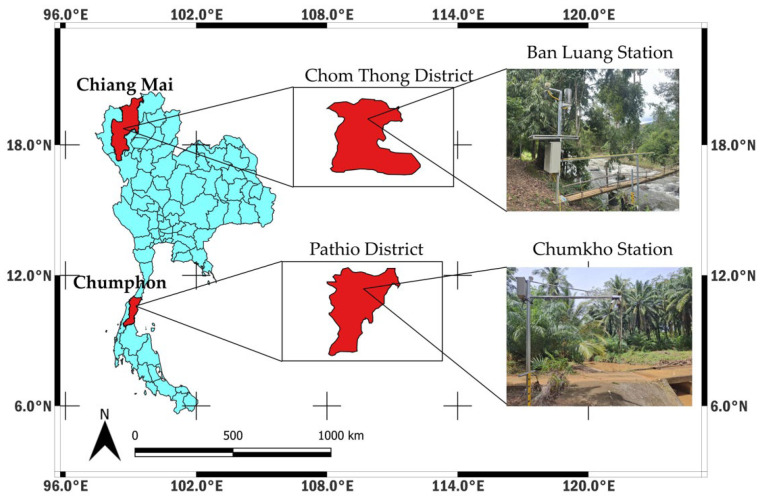
Geographic locations of the two experimental deployment sites for the proposed AIoT-based early flash-flood warning system.

Figure 6 shows the locations of the two field deployment sites used to evaluate the AIoT-based early flash-flood warning system. The experimental study was conducted in two hydrologically distinct regions of Thailand: the Chom Thong district in Chiang Mai province, northern Thailand, and the Pathio district in Chumphon province, southern Thailand, to validate the system under diverse climatic and environmental conditions.

The upper panel of Figure 6 shows the Ban Luang station in the Chom Thong district, Chiang Mai, located at 18.28° N, 98.36° E, as shown on QGIS maps. The site lies within a mountainous watershed with steep river gradients and rapid runoff, making it highly susceptible to flash floods during intense rainfall. The installed prototype comprises a staff gauge, a CCTV monitoring system, and a multi-sensor unit positioned along a narrow, fast-flowing stretch of river.

The lower panel shows the Chumkho station in the Pathio district, Chumphon province, located at 10.79° N, 99.34° E, as shown on a QGIS map. Unlike the northern site, the southern region features lowland floodplains with dense vegetation and a slower river flow. The installed monitoring system captures hydrological variations in a palm plantation area where flash floods often develop rapidly due to upstream rainfall and soil saturation.

By deploying the prototype in two hydrologically contrasting environments, we assess the AIoT-based system’s robustness, adaptability, and generalization under real-world conditions, thereby validating its effectiveness for rural flood-resilience applications.

**Figure 7:**
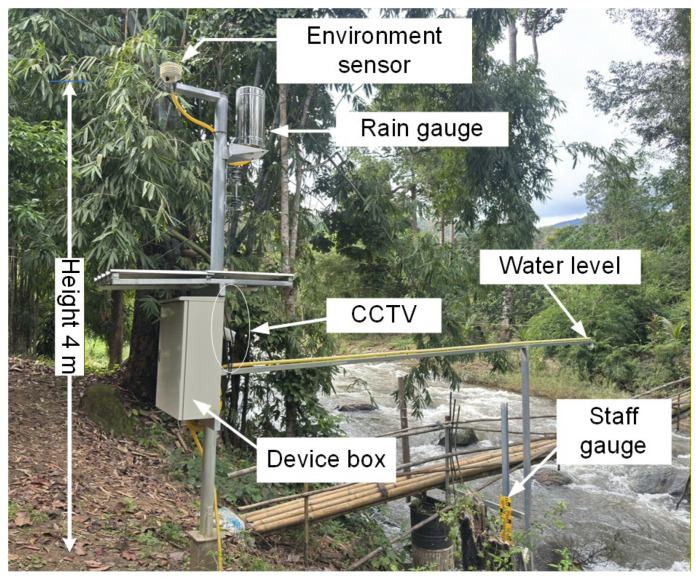
The test site at the Ban Luang station in Chom Thong District, Chiang Mai province.

Figure 7 shows the fully deployed site station in Ban Luang, Chom Thong district, Chiang Mai Province. An environmental sensor is mounted at a height of 4 m to measure atmospheric variables, including air temperature, PM_2.5_ particulate matter, and humidity. A tipping-bucket rain gauge measures rainfall intensity at high temporal resolution. A CCTV camera is strategically positioned to monitor the staff gauge installed along the riverbank, enabling the YOLOv10 model to perform continuous visual water-level detection under varying illumination and weather conditions. An ultrasonic water-level sensor is mounted horizontally across the river channel to measure real-time water depth, providing ground-truth hydrological data for LSTM forecasting. All sensing devices connect to the device box, which houses the AIoT edge server, power management system, and communication modules. This configuration enables the station to operate autonomously, to process data locally, and to transmit results to the cloud platform for alert generation. The Ban Luang station serves as a critical testbed for evaluating the system’s performance in high-variability, high-velocity mountain streams.

**Figure 8:**
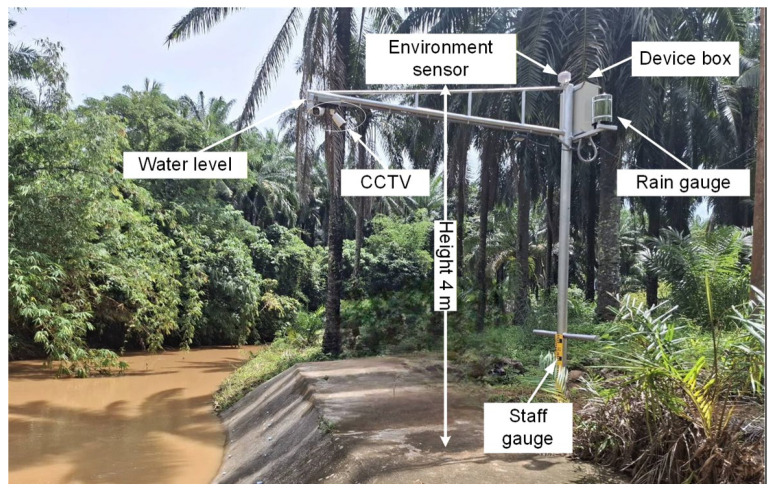
The test site at the Chumkho station in the Pathio district, Chumphon province.

Figure 8 shows the deployed monitoring system at the Chumkho station in the Pathio district, Chumphon. This site represents a lowland tropical catchment with flat terrain, dense vegetation, sediment-laden water, and relatively slow-flowing channels. Unlike the mountainous Ban Luang station, flood events in this region are typically driven by prolonged rainfall, upstream flow accumulation, and tidal interactions during peak monsoon periods. The station features an environmental sensor at the top that records water level, precipitation, light intensity, brightness, temperature, and relative humidity. A tipping-bucket rain gauge is mounted near the device box to quantify real-time rainfall intensity. An ultrasonic water-level sensor is mounted horizontally in the river channel to measure water-stage variations at high temporal resolution. A CCTV camera installed directly beneath the mast captures visual footage of the staff gauge, enabling the YOLOv10 model to detect river-level changes in real time. All sensors connect to the device box, which houses the AIoT edge server, power supply, and communication modules that relay the processed data to the cloud computing platform. In the current implementation, the system is powered by a stable grid supply; however, the proposed architecture is designed to be compatible with renewable energy sources. In particular, solar photovoltaic (PV) systems with battery storage can be integrated to support off-grid operation. The low-power design of the IoT sensors and edge server (typically lower than 50 W) makes the system suitable for solar-based deployment.

### 5.2. Dataset Description

The evaluation used two complementary datasets: first, a CCTV-based image dataset for YOLOv10 visual flood detection, and second, a multivariate hydrological time-series dataset for LSTM forecasting. Both datasets were collected at two rural monitoring sites, namely the Ban Luang station in the Chom Thong district, Chiang Mai, and the Chumkho station in the Pathio district, Chumphon. These sites were selected to represent distinct hydrological conditions, enabling a comprehensive assessment of model robustness across mountainous and lowland environments.

#### 5.2.1. Image Dataset for YOLOv10

A total of 1200 images were collected from IP CCTV cameras installed at the two monitoring stations under diverse environmental and hydrological conditions, including variations in lighting, weather, and flow regime. Each image was manually annotated with staff-gauge bounding lines, water-level positions, and corresponding flood-state labels. To enhance model generalization, the images were resized to a uniform resolution and augmented with random flips, brightness adjustments, noise injection, and rain simulation. The dataset was then split into training, validation, and testing subsets. The complete specification of the image dataset and the associated training configuration is summarized in Table 3.

#### 5.2.2. Hydrological Time-Series Dataset for LSTM

The multivariate time-series dataset was acquired from environmental and hydrological IoT sensors deployed at both stations. The sensor suite recorded six physical variables relevant to flash-flood dynamics at a uniform 1-h sampling interval over a 60-day monitoring period, yielding 2942 valid time steps for the LSTM model training and evaluation. The measured variables and the dataset specifications are listed in Table 4.

Table 4 summarizes the hyperparameter configuration of the proposed LSTM model used for short-term water-level forecasting. The model utilizes a two-layer stacked LSTM architecture with 128 and 64 hidden units to capture temporal dependencies in hydrological observations. A 24-time-step look-back window was selected to represent the previous 24 h of environmental conditions, while a 1-h forecasting horizon was adopted to support early warning applications.

### 5.3. Detection Metrics for YOLOv10

YOLOv10 is designed for object detection and classification tasks. Therefore, its performance is evaluated using standard detection metrics, including precision, recall, and mean average precision (mAP).

- Precision:

Precision is the proportion of correctly detected objects among all detections and is expressed as follows:

(12) $$\mathrm{Precision}=\frac{TP}{TP+FP}$$

where TP is true positive when correctly detecting at staff gauge and FP is the false positive when there is incorrect detection of CCTV at the staff gauge.

- Recall:

Recall evaluates the model’s ability to detect all relevant objects as follows:

(13) $$\mathrm{Recall}=\frac{TP}{TP+FN}$$

where FN is the false negative or missed staff-gauge detections.

- Mean average precision (mAP):

Average precision (AP) is the area under the precision–recall curve for a given class. The mAP is defined as follows:

(14) $$\mathrm{mAP}=\frac{1}{N}\sum_{i=1}^{N}AP_{i}$$

where N is the number of image dataset class alerts (N = 1200), such as low-level, mid-level, and high-level and AP is the average precision. We note that mAP = 0.5 is reported, meaning that a detection is considered correct if the intersection over union (IoU) between the predicted and ground-truth bounding lines exceeds 0.5.

### 5.4. Regression Metrics for LSTM Water Level Prediction

The LSTM model performs continuously-valued time-series forecasting. Its performance is evaluated using regression metrics, including the root mean square error (RMSE), the mean absolute error (MAE), and the coefficient of determination ($R^{2}$). We define $y_{true}$ as the observed water level and $y_{pre}$ as the predicted water level at time t.

- RMSE:

The RMSE penalizes large prediction errors and reflects the overall forecasting accuracy. The RMSE can be expressed as follows:

(15) $$\mathrm{RMSE}=\sqrt{\frac{1}{M}\sum_{t=1}^{M}{\left(y_{true}-y_{pre}\right)}^{2}}$$

where M is the number of training samples.

- MAE:

The MAE represents the average absolute difference between the predicted and observed values. It provides an intuitive measure of average prediction error in water-level units (meters). The MAE can be expressed as follows:

(16) $$\mathrm{MAE}=\frac{1}{M}\sum_{t=1}^{M}\left(y_{true}-y_{pre}\right)$$

- Coefficient of Determination ($R^{2}$):

The $R^{2}$ metric evaluates the proportion of variance in the observed data explained by the LSTM model as follows:

(17) $$R^{2}=1-\frac{\sum_{t=1}^{M}{\left(y_{true}-y_{pre}\right)}^{2}}{\sum_{t=1}^{M}{\left(y_{true}-{\bar{y}}_{true}\right)}^{2}}$$

where ${\bar{y}}_{true}$ is the mean of observed water levels.

### 5.5. Alert Threshold and Fusion Logic

To improve operational reliability and to reduce false alarms, the proposed AIoT-based flash-flood warning system employs a hybrid decision-making mechanism that integrates sensor measurements, LSTM-based forecasting, and YOLOv10-based visual detection. Instead of relying on a single sensing source, multiple information streams are fused to generate warning decisions for rural flood monitoring.

The warning system defines three operational alert levels, low, medium, and high, based on the water-level thresholds calibrated using staff-gauge observations and local field measurements. The warning state at time t is defined as follows:

(18) $$A_{t}=\left\{\begin{array}{ll} low, & H_{sens}\left(t\right)<T_{1}\\ meduim, & T_{1}\leq H_{sens}\left(t\right)<T_{2}\\ high, & H_{vis}\left(t\right)\geq T_{2} \end{array}\right.$$

where $H_{sens}\left(t\right)$ is the water level from the ultrasonic sensor, $H_{vis}\left(t\right)$ is the water level detection from CCTV camera, $T_{1}$ denotes the medium-risk threshold, and $T_{2}$ denote the high-risk threshold.

For the deployed field stations, the threshold values are selected through site-specific operational calibration. Table 5 shows the water level warning thresholds.

The fusion score is calculated as follows:

(19) $$R_{t}=w_{1}S_{sens}+w_{2}S_{LSTM}+w_{3}S_{YOLO}$$

where $S_{sens}$ denotes the ultrasonic sensor warning state, $S_{LSTM}$ denotes the predicted warning state, $S_{YOLO}$ denotes the visual detection state, and $w_{1}$, $w_{2}$, $w_{3}$ are weighting coefficients.

In practice, a high-risk alert is triggered when

(20) $$R_{t}\geq T_{2}$$

**Figure 9:**
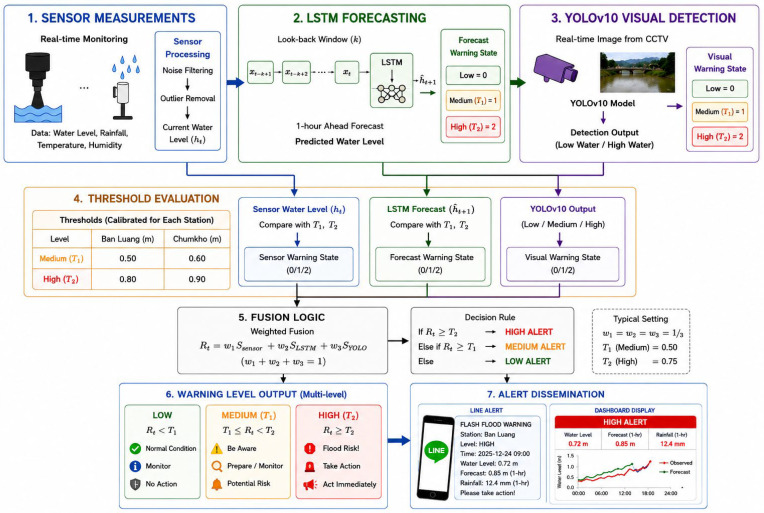
Hybrid decision process integrating sensor measurements, LSTM forecasting, YOLOv10 visual detection, threshold evaluation, and weighted fusion logic for multi-level flood warning generation and alert dissemination.

Figure 9 illustrates the workflow of the proposed AIoT-based early flash-flood warning system. The framework integrates real-time sensing, LSTM-based prediction, YOLOv10-based visual verification, and decision-fusion mechanisms to generate reliable multi-level warning information for rural disaster resilience.

The first stage consists of sensor measurements and preprocessing, where hydrological and environmental parameters, including water level, rainfall, temperature, and humidity, are continuously collected from field sensors. In the next stage, an LSTM forecasting model is employed to predict future water-level conditions using historical observations within a predefined look-back window.

Simultaneously, a YOLOv10-based visual detection module processes real-time CCTV images to monitor water levels. The threshold evaluation stage compares three information sources, including current sensor measurements, LSTM forecasting outputs, and YOLOv10 visual detection results. For the Ban Luang station, the medium- and high-risk thresholds were set at 0.50 m and 0.80 m, respectively; whereas, at the Chumkho station, the thresholds were 0.60 m and 0.90 m, respectively.

Next stage, the resulting fusion score ($R_{t}$) is mapped into low-, medium-, and high-risk warning levels. Low-risk conditions indicate normal operation; medium-risk conditions suggest increased awareness and monitoring; and high-risk conditions trigger immediate emergency actions.

Finally, the warning results are disseminated via LINE notifications and dashboard visualizations, enabling community members and local authorities to gain real-time situational awareness and to receive rapid warning information.

## 6. Result

### 6.1. AIoT Performance

This section presents the results of the AIoT-based early flash-flood warning system. We demonstrate that the collected datasets support effective visual CCTV detection, hydrological forecasting, and a real-time warning application.

**Figure 10:**
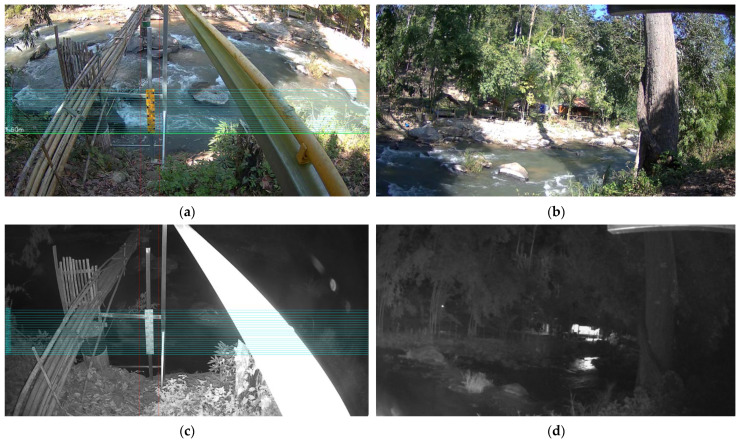
Comparison of visual water-level monitoring using CCTV cameras under different lighting conditions at the Ban Luang station: (**a**) CCTV with YOLOv10 detection during daylight; (**b**) Conventional CCTV monitoring during daylight; (**c**) CCTV with YOLOv10 detection during nighttime; (**d**) Conventional CCTV monitoring during nighttime.

Figure 10a–d show effective visual CCTV detection using YOLOv10 and conventional monitoring under both daylight and nighttime conditions at the Ban Luang station. The results clearly demonstrate that YOLOv10 enables continuous, automated, and lighting-invariant visual water-level detection, outperforming conventional CCTV monitoring that relies on manual observation.

Figure 10a shows YOLOv10 operating for multiple-line water-level detection at the staff gauge. The initial water level is 0.5 m, the difference between the normal river water level and the staff gauge level. YOLOv10 detects this initial level to alert of the rising river water levels and to warn of the maximum level when the water surface reaches the upper edge of the staff gauge.

**Figure 11:**
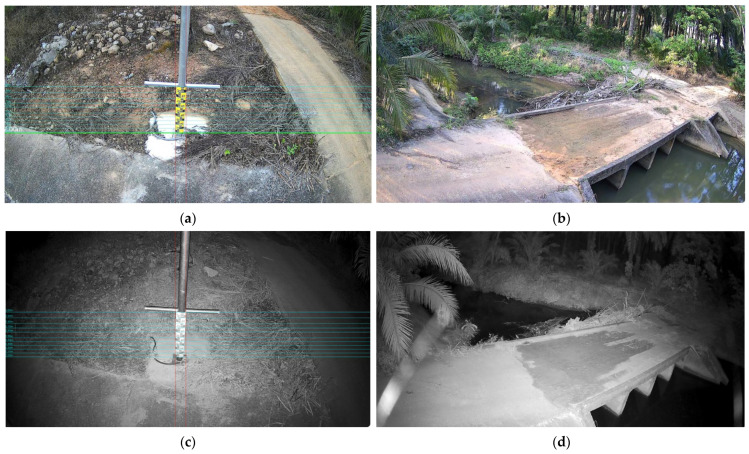
Comparison of visual water-level monitoring using CCTV cameras under different lighting conditions at the Chumkho station: (**a**) CCTV with YOLOv10 detection during daylight; (**b**) Conventional CCTV monitoring during daylight; (**c**) CCTV with YOLOv10 detection during nighttime; (**d**) Conventional CCTV monitoring during nighttime.

Figure 11a–d compare visual water-level monitoring using CCTV cameras at the Chumkho station under different lighting conditions, both during daylight and at night. A total of 10 boundary lines in YOLOv10 are annotated for water-level detection from the staff gauge.

**Figure 12:**
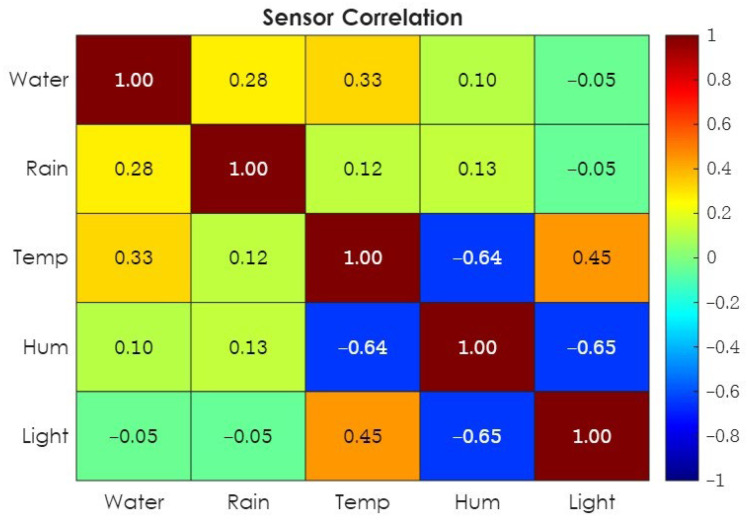
Sensor correlation matrix for the dataset collected at the Ban Luang station in Chiang Mai from 24 October to 24 December 2025.

**Figure 13:**
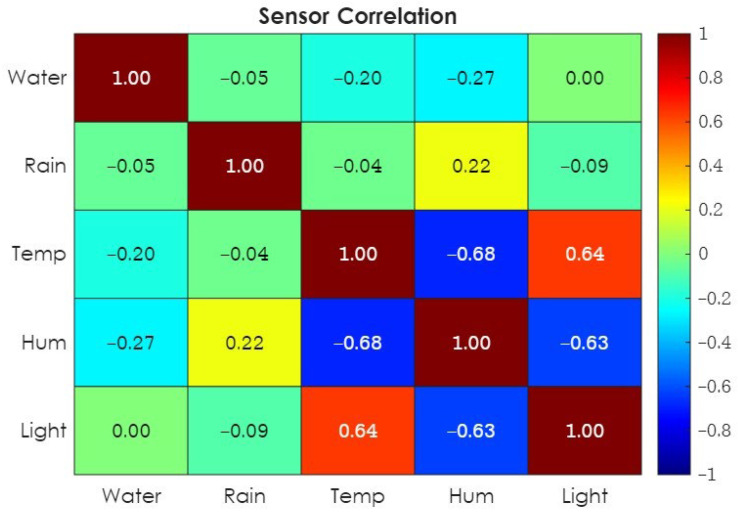
Sensor correlation matrix for the dataset collected at the Chumkho station in Chumphon from 24 October to 24 December 2025.

Figure 12 and Figure 13 show a correlation matrix heatmap of the environmental sensor data collected at the Ban Luang station in the Chom Thong district, Chiang Mai, and the Chumkho station in the Pathio district, Chumphon. The matrix visualizes linear relationships among the hydrological parameters, including water level, rainfall, temperature, humidity, and light intensity. The color scale ranges from −1.0 (blue) to +1.0 (red), indicating perfect negative and perfect positive correlations, respectively. Strong inverse relationships are observed between temperature and humidity, whereas positive correlations exist between rainfall intensity and subsequent water-level rise.

**Figure 14:**
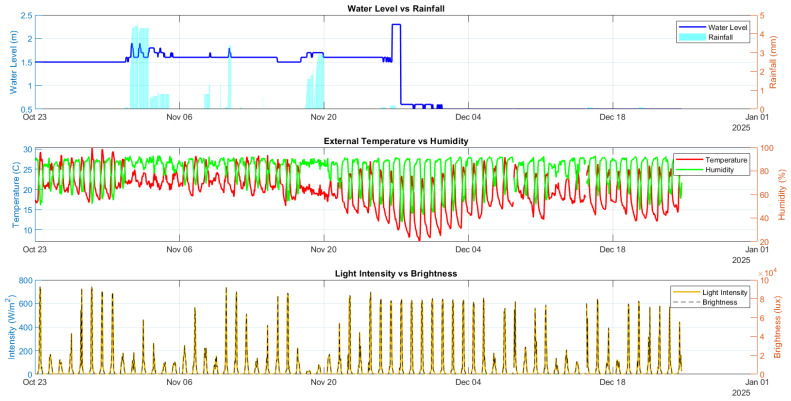
Time series of the dataset from 24 October to 24 December 2025, at the Ban Luang station in Chiang Mai.

**Figure 15:**
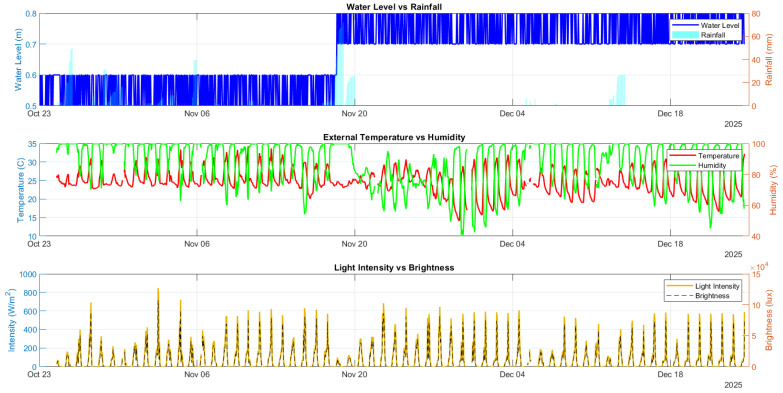
Time series of the dataset from 24 October to 24 December 2025, at the Chumkho station in Chumphon.

Figure 14 and Figure 15 show the temporal evolution of hydrological and meteorological time series at the Ban Luang and Chumkho stations, respectively, revealing distinct system dynamics across different geographical and hydrological settings. At the Ban Luang station in Figure 13, the water-level time series shows rapid, pronounced increases after rainfall events, indicating a flashy hydrological response typical of mountainous catchments with steep slopes and limited storage capacity. These abrupt rises, together with short lag times between rainfall and water-level peaks, underscore the critical need for rapid detection and short-term forecasting in upland, flood-prone areas.

By contrast, the Chumkho station in Figure 15 shows more buffered, stable water-level behavior, with variations confined to a narrower range despite intermittent rainfall. This response reflects the characteristics of a coastal lowland environment, in which a flatter terrain, greater infiltration capacity, and channel storage attenuate rapid runoff. The observed step-like rise in the water level during sustained rainfall periods suggests gradual accumulation rather than sudden surges, underscoring the importance of capturing longer-term dependencies in forecasting models.

Across both stations, temperature, humidity, and light-related variables show consistent diurnal cycles, confirming reliable sensor operation and strong atmospheric forcing. The inverse relationship between temperature and humidity, along with the strong daily periodicity of light intensity and brightness, provides valuable contextual information that complements the rainfall measurements.

In practice, YOLOv10-only CCTV detection was compared qualitatively with ultrasonic sensor-based water-level readings. The sensor provides continuous numerical water-level values, while YOLOv10 provides visual confirmation of low- and high-water states from the staff gauge. The integration of the two modalities enhances alert reliability, especially under turbulent or low-visibility conditions.

**Figure 16:**
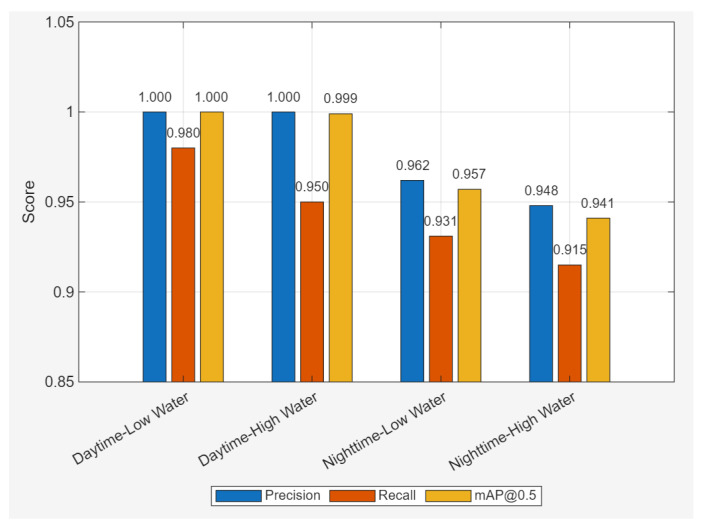
YOLOv10 performance score at the Ban Luang station under daytime, nighttime, low-water, and high-water conditions.

Figure 16 shows the YOLOv10 performance score at the Ban Luang station under different environmental conditions, including daytime, nighttime, low water, and high-water scenarios, as listed in Table 6. The experimental evaluation demonstrated that the proposed visual detection framework maintained high precision, recall, and mAP@0.5 performance under all operating conditions. The highest performance was observed during daytime and low-water conditions, where the model achieved nearly perfect detection accuracy. Slight performance degradation occurred during nighttime and high-water conditions due to reduced illumination. Nevertheless, the YOLOv10 framework continued to provide reliable visual flood detection capability for practical flood monitoring applications.

**Figure 17:**
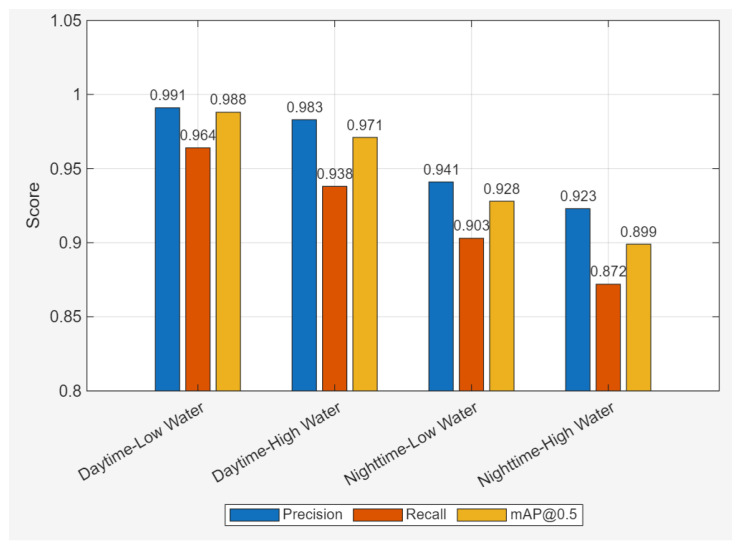
YOLOv10 performance score at the Chumkho station under daytime, nighttime, low-water, and high-water conditions.

Figure 17 shows the YOLOv10 performance score at the Chumkho station under different environmental conditions. The highest performance was achieved during daytime and low-water conditions, with precision, recall, and mAP@0.5 values of 0.991, 0.964, and 0.988, respectively. The performance slightly decreased during high-water and nighttime conditions due to reduced illumination, rainfall disturbance, reflection effects, and rapidly varying coastal hydrodynamic conditions. The lowest performance was observed at night and under high-water conditions, with recall and mAP@0.5 decreasing to 0.872 and 0.899, respectively. Despite these challenges, the YOLOv10 framework maintained relatively high detection accuracy under all evaluated conditions, demonstrating its suitability for practical flood monitoring applications.

Table 6 and Table 7 summarize the YOLOv10 performance scores at Ban Luang station and Chumkho station under different environmental conditions. 

**Figure 18:**
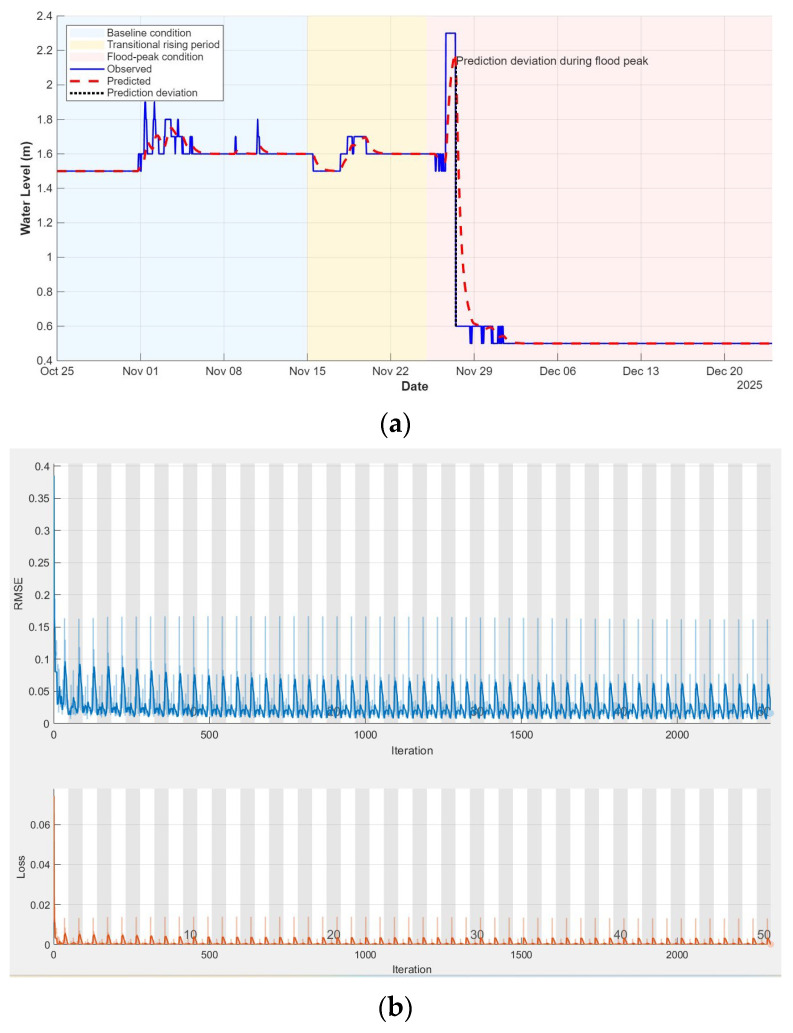
LSTM prediction for the Ban Luang station, based on the dataset collected from 24 October to 24 December 2025: (**a**) Predicted water level; (**b**) RMSE and loss at the maximum of 2300 iterations.

**Figure 19:**
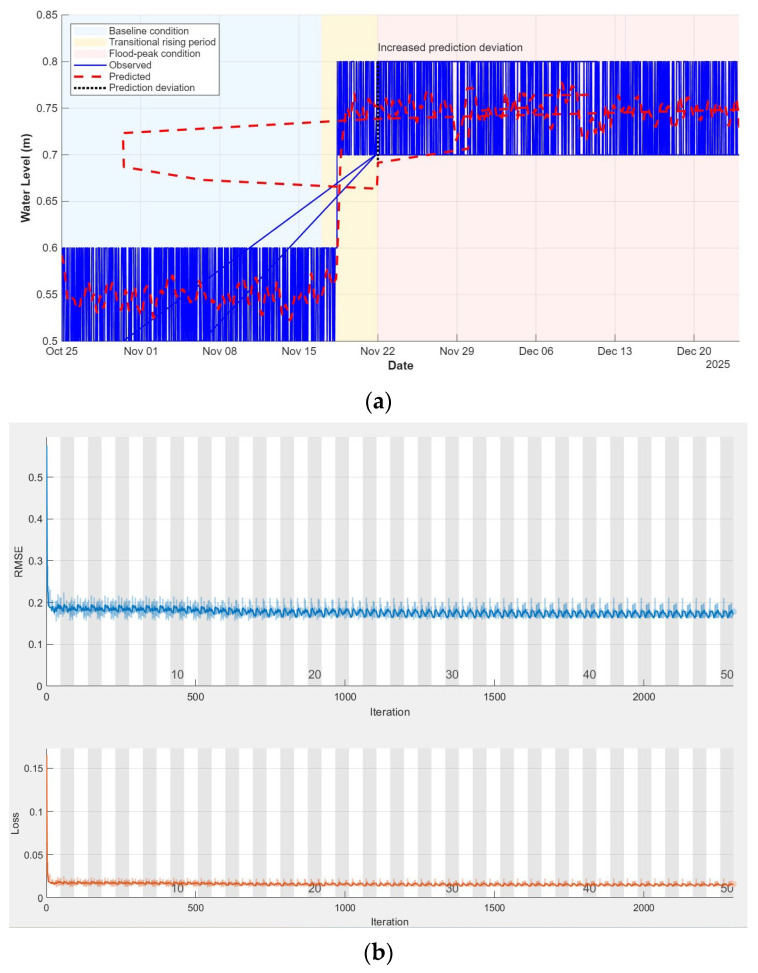
LSTM prediction for the Chumkho station, based on the dataset collected from 24 October to 24 December 2025: (**a**) Predicted water level; (**b**) RMSE and loss at the maximum of 2300 iterations.

Figure 18a,b and Figure 19a,b show water-level forecasts from an LSTM model for the Ban Luang and Chumkho stations, respectively. The results are presented as the RMSE and loss over iterations. The training cycle used 50 epochs, a learning rate of 0.01, and a maximum of 2300 training iterations. Additionally, the LSTM prediction performance for both stations is shown in Table 8.

The LSTM model was configured with a look-back window k of 24 samples, representing the previous 24 h of hydrological and environmental observations. The forecast horizon was set to 1 time step, corresponding to a forecast horizon $H_{sens}$ 1 h-ahead water-level prediction. Since the dataset was recorded at hourly intervals, shorter horizons, such as 10–30 min, were not evaluated in this study. Thus, the LSTM results represent the 1 h-ahead forecasting performance.

Table 8 summarizes the error decomposition analysis of the LSTM prediction performance at the Ban Luang and Chumkho stations under different hydrological conditions. The analysis was performed by separating the monitoring period into baseline, transitional rising-water, and flood-peak conditions to investigate the temporal distribution of forecasting error. The results indicate that the Ban Luang station achieved a consistently stable forecasting performance throughout all hydrological conditions. The R^2^ values remained above 0.97 for all conditions, while the RMSE and MAE values remained relatively low. Although prediction deviation slightly increased during flood-peak periods, the LSTM model continued to accurately track the temporal variation of water-level behavior.

By contrast, the Chumkho station exhibited larger forecasting errors during transitional and flood-peak conditions. Under baseline conditions, the model still achieved a relatively strong performance with an R^2^ value of 0.912. However, the prediction error increased significantly under rapidly changing hydrological conditions, with the RMSE rising to 0.094 m and the R^2^ decreasing to 0.693 during flood-peak events.

The results confirm that the reduced overall forecasting performance at the Chumkho station primarily originated from rapid transitional and flood-peak fluctuations rather than from stable baseline conditions. The increased prediction deviation was likely attributable to non-linear hydrodynamic effects, including tidal interactions, backwater effects, rainfall-runoff variability, and abrupt coastal water-level oscillations. Despite these challenges, the LSTM framework remained capable of providing operationally useful short-term forecasting information for flood early warning applications.

### 6.2. Alert Event Validation

The alert event plays a critical role in converting the outputs of the proposed AIoT-based flash-flood monitoring framework into actionable information for local communities. By integrating real-time outputs from the YOLOv10 object detection module and an LSTM-based forecasting model, the alert application enables timely, context-aware dissemination of flood warnings to end users via mobile platforms.

**Figure 20:**
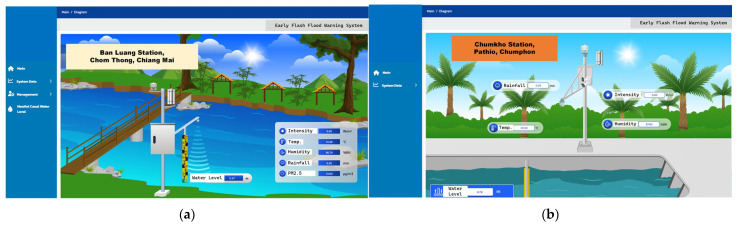
Web-based alert application on mobile platforms: (**a**) Alert application interface for the Ban Luang station, Chom Thong district, Chiang Mai; (**b**) Alert application interface for the Chumkho station, Pathio district, Chumphon.

Figure 20a,b show the operational alert application interface for the proposed AIoT-based early flash-flood warning system, available on the web-based mobile platform at https://ws1.saijai.tech/diagram (accessed on 24 December 2025). The interface serves as the final interaction layer between the underlying AIoT models and end users, translating complex sensor measurements and AIoT-derived insights into intuitive, real-time situational awareness for rural communities.

Figure 20a shows the dashboard application for the Ban Luang station in the Chom Thong district, Chiang Mai, while Figure 20b shows the Chumkho station in the Pathio district, Chumphon. Both interfaces use map-based and schematic visualizations that reflect the physical layout of each monitoring site, including river geometry, bridges, and sensor installation points. This contextual visualization enables users to immediately map numerical values to real-world locations, thereby enhancing interpretability and trust in the system.

The dashboard application displays real-time environmental and hydrological parameters, including water level, rainfall, temperature, humidity, and light intensity, continuously updated from the AIoT edge server and cloud platform. Water-level indicators are visually emphasized, enabling a rapid assessment of the current flood risk. When combined with outputs from the YOLOv10 visual detection module and an LSTM-based forecasting model, the application supports both real-time monitoring of current conditions and anticipatory awareness of potential flash-flood events.

Alert events were validated using staff-gauge measurements and CCTV-assisted visual confirmation. Event-level performance was evaluated using true positives (TP), false positives (FP), false negatives (FN), false alarm ratio (FAR), probability of detection (POD), and critical success index (CSI). These metrics provide a quantitative assessment of alert reliability beyond model prediction accuracy.

The FAR, POD, and CSI are expressed as follows:

(21) $$FAR=\frac{FP}{TP+FP}$$

(22) $$POD=\frac{TP}{TP+FN}$$

(23) $$CSI=\frac{TP}{TP+FP+FN}$$

Table 9 shows the event-level alert validation performance during 24 October–24 December 2025, the proposed system reported 45 alert events across the Ban Luang and Chumkho stations. Of these, 40 events were verified as TP, 5 as FP, and 3 as FN.

The results indicate an overall FAR of 0.111, a POD of 0.930, and a CSI of 0.833, demonstrating that the hybrid YOLOv10–LSTM framework provides reliable operational alert performance while maintaining a low false-alarm rate.

The event-level analysis demonstrated that integrating predictive time-series modeling with visual confirmation significantly improves the reliability of early flash-flood warning systems and reduces the likelihood of false alarms in operational practice.

**Figure 21:**
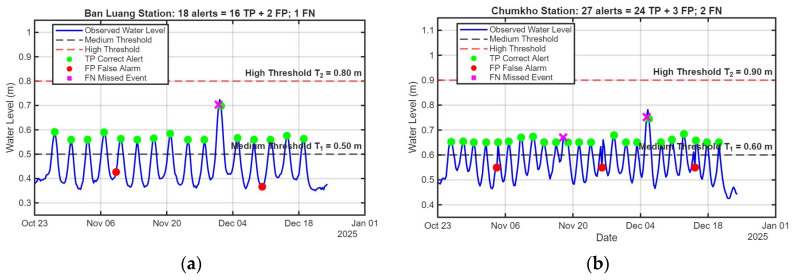
Event-level water-level alert validation results: (**a**) The Ban Luang station; (**b**) The Chumkho station during the monitoring period from 24 October to 24 December 2025. Green markers indicate TP alerts, red markers indicate FP alerts, and magenta markers indicate FN events.

Figure 21a shows the alert-event performance at the Ban Luang station. The measured water level generally fluctuated around the medium-risk threshold $T_{1}$ of 0.50 m, while one significant event approached the high-risk threshold $T_{2}$ of 0.80 m. The system reported a total of 18 alert events, including 16 TP, 2 FP, and 1 FN. Most TP events occurred when the water level approached the medium threshold, indicating that the proposed fusion framework successfully identified rising flood conditions. The two FP events occurred during transitional water-level fluctuations slightly below the warning threshold, suggesting that the forecasting and visual detection modules occasionally issued precautionary warnings under uncertain hydrological conditions. One FN event occurred during a rapid rise in water level and was not immediately classified as a warning condition. Nevertheless, the overall event-detection performance remained highly reliable.

Figure 21b illustrates the corresponding results at the Chumkho station. Compared with Ban Luang, the Chumkho station exhibited more frequent water-level oscillations and more warning events. The proposed system reported 27 alert events, including 24 TP, 3 FP, and 2 FN. The majority of TP events were correctly identified when they exceeded the medium threshold $T_{1}$ of 0.60 m. The FP events mainly occurred during short-term fluctuations near the threshold boundary, where LSTM predictions and YOLOv10 visual confidence indicated an increasing flood risk, even though the measured water level did not fully meet the predefined warning condition. The FN events were associated with abrupt water-level transitions and temporary inconsistencies between sensor measurements and visual confirmation.

The results demonstrated that the proposed hybrid framework, which combines sensor measurements, LSTM forecasting, and YOLOv10 visual verification, can effectively identify flood-related warning events under practical field conditions. The TP events substantially outnumbered the FP and FN events at both stations, indicating favorable operational reliability and a relatively low false-alarm rate. The event-level analysis further confirms that integrating predictive forecasting with visual confirmation improves the robustness of warnings compared with conventional threshold-only monitoring approaches.

Table 10 summarizes the representative fusion-score results for water-level alert events during the monitoring period from 24 October to 24 December 2025. The fusion score combines the following three normalized sources of information: current water-level status from the ultrasonic sensor, short-term water-level prediction from the LSTM model, and visual confirmation from YOLOv10-based CCTV detection. The results show that true-positive alert events consistently produced high fusion scores, with mean values of 0.857 at the Ban Luang station and 0.840 at the Chumkho station, indicating high-level warnings.

False-positive events produced moderate fusion scores of 0.503 and 0.513, indicating that alerts were mainly triggered during transitional conditions where either the LSTM forecast or YOLOv10 visual confidence suggested an increasing risk, but the hydrological threshold was not subsequently confirmed. False-negative events showed medium-level fusion scores because the sensor and LSTM outputs indicated rising water levels, while YOLOv10 confidence was relatively low, likely due to visual obstruction, rainfall, turbulence, or poor lighting conditions.

Overall, TP events achieved a mean fusion score of 0.850, while FP and FN events remained in the medium-risk range. This demonstrates that the proposed fusion strategy effectively distinguishes high-confidence warning conditions from uncertain transitional events, thereby supporting more reliable early flash-flood alert generation.

### 6.3. End-to-End Latency Performance

The operational performance of the proposed AIoT-based early flash-flood warning system was further evaluated through an end-to-end (E2E) latency analysis during field deployment at the Ban Luang and Chumkho stations, available at the link http://152.42.170.95:8085/ (accessed on 24 December 2025). The latency analysis included the following four primary processing stages: sensor acquisition and transmission, edge AI processing, cloud synchronization, and mobile notification delivery.

The total E2E latency is expressed as follows:

(24) $$T_{Total}=T_{sens}+T_{edge}+T_{cloud}+T_{notify}$$

where $T_{sens}$ denotes the latency associated with sensor acquisition and data transmission from the field device, $T_{edge}$ represents the edge AI processing latency, including YOLOv10 and LSTM inference, $T_{cloud}$ corresponds to cloud/database synchronization latency, and $T_{notify}$ represents the delay associated with notification delivery to the user.

Table 11 summarizes the E2E latency performance. The results indicate that the Ban Luang station achieved a minimum latency of 3.08 s, a maximum of 6.28 s, and an average of 4.41 s, with a standard deviation of 0.80 s. The relatively low standard deviation demonstrates a stable communication and processing performance throughout the monitoring period. For the Chumkho station, the minimum latency was 2.51 s, the maximum was 8.89 s, the average was 4.73 s, and the standard deviation was 1.19 s. The E2E latency performance confirms that the proposed AIoT framework can support near real-time flood early-warning applications in rural environments.

**Figure 22:**
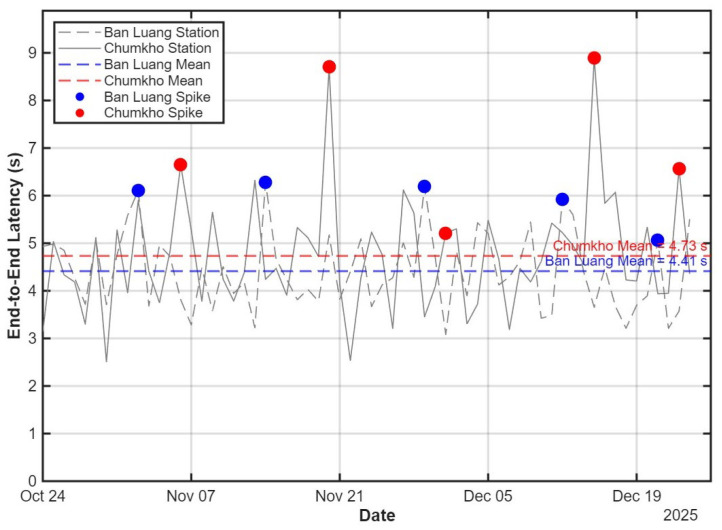
Total E2E latency performance of the proposed AIoT-based flood warning system at the Ban Luang and Chumkho stations during the monitoring period.

Figure 22 shows the E2E latency performance of the proposed AIoT-based flood warning framework during field deployment at the Ban Luang and Chumkho stations from 24 October to 24 December 2025. At the Ban Luang station, the average E2E latency was 4.41 s, while at the Chumkho station it was 4.73 s. The increased latency observed at the Chumkho station was primarily due to greater communication variability and cloud synchronization delays under coastal environmental conditions. Nevertheless, both stations maintained latency below approximately 5 s under normal operating conditions, demonstrating the proposed framework’s capability for near real-time warning dissemination.

Several temporary latency spikes were observed during the monitoring period, as indicated by the highlighted markers in Figure 22. At the Ban Luang station, several peak latency events exceeded 6 s, while the Chumkho station experienced occasional latency peaks approaching 9 s. These transient increases were primarily attributed to temporary 5G communication instability, cloud synchronization overhead, and network congestion during periods of heavy rainfall and intensive data transmission.

During the 60-day deployment period, the proposed AIoT-based warning framework maintained high operational availability under practical environmental conditions. The Ban Luang station achieved approximately 98.9% system uptime, while the Chumkho station achieved approximately 97.8% uptime despite experiencing more variable communication conditions during heavy rainfall events.

Several temporary communication interruptions were observed during the monitoring period, particularly during periods of elevated rainfall intensity and unstable network conditions. The longest outage durations were approximately 12 min at the Ban Luang station and 21 min at the Chumkho station. However, no permanent data loss occurred because the edge-computing platform locally buffered the sensor measurements and AI inference outputs during the temporary outages and automatically synchronized the stored data with the cloud platform upon the recovery of communication.

The deployed framework also employed backup battery support to maintain short-duration operational continuity during temporary power fluctuation events. In addition, the automatic cloud resynchronization mechanism improved system robustness and minimized data gaps during periods of intermittent communication. These results demonstrate the practical operational resilience of the developed AIoT platform for real-time rural flood monitoring applications. Table 12 presents the system resilience and operational reliability for the 60-day period from 24 October to 24 December 2025.

### 6.4. Community-Level Deployment and Alert Dissemination

The proposed AIoT-based flood warning system was deployed and evaluated under practical field conditions at the Ban Luang and Chumkho stations. In addition to sensor and AI model validation, the system was connected to a real-time mobile notification platform via the LINE application to support last-mile warning dissemination to local communities.

The warning interface employed simplified operational states consisting of low-, medium-, and high-risk levels to improve usability and interpretability for non-technical users. The dashboard interface displayed current water-level conditions, warning status, CCTV monitoring, and alert notifications through a graphical visualization platform accessible via smartphones and web-based interfaces.

During the deployment period, warning notifications were transmitted to participating local stakeholders, community volunteers, and local administrative personnel through the LINE messaging platform. The mobile-based dissemination mechanism enabled users to receive warning information in real time without requiring specialized monitoring equipment. The use of smartphones and commonly available internet connectivity improved accessibility for rural deployment conditions.

Informal operational observations during the deployment period indicated that the warning dashboard and mobile notification system improved situational awareness among local users, particularly during periods of elevated rainfall and rising water levels. The simplified warning structure and mobile accessibility were found suitable for practical community-level deployment.

## 7. Discussion

The proposed AIoT-based early flash-flood warning system, which integrates YOLOv10 visual detection and LSTM-based hydrological forecasting, delivers reliable, complementary performance at two geographically distinct rural stations, Ban Luang and Chumkho. The deployment at two contrasting sites (Ban Luang is mountainous upstream, and Chumkho is lowland downstream) was intentionally designed to evaluate the system’s robustness under different hydrological regimes.

Considering YOLOv10’s performance in visual water-level detection at both stations, it consistently achieved high detection accuracy under both low- and high-water conditions. Precision remained at 1.000, indicating an extremely low false-alarm rate, which is critical for operational early-warning systems. Recall decreased slightly during high-water conditions at both sites, dropping from 0.980 to 0.950 at Ban Luang and from 0.970 to 0.937 at Chumkho. This reduction is primarily due to increased visual complexity from surface turbulence, debris, reflections, and partial occlusion of the staff gauge under elevated flow conditions. Nevertheless, the mAP@0.5 values remained above 0.999, confirming robust localization performance.

Compared with conventional CCTV monitoring, YOLOv10 enables automated, quantitative interpretation of visual data, thereby significantly improving reliability in nighttime and adverse weather conditions. These results show that CCTV cameras, when augmented with YOLOv10, can function as intelligent visual sensors rather than passive monitoring tools.

The YOLOv10 framework performs binary visual classification consisting of low-water and high-water conditions. The intermediate medium-level warning state is generated during fusion and threshold evaluation by combining sensor measurements, LSTM forecasting outputs, and visual detection results, using predefined warning thresholds.

For LSTM-based water-level forecasting, the model performed well at the Ban Luang station, achieving an RMSE of 0.057 m, an MAE of 0.015 m, and an R^2^ of 0.987. It accurately captured both the gradual variations and abrupt changes in water level associated with rainfall-driven events, reflecting the site’s relatively stable hydrology and upstream conditions.

At the Chumkho station, the error decomposition analysis showed that most forecasting errors occurred during transitional and flood-peak conditions rather than during stable baseline periods. Under baseline conditions, the LSTM model achieved a relatively stable forecasting performance with an R^2^ exceeding 0.9. However, prediction deviation increased significantly during rapid water-level transitions and flood-peak events characterized by abrupt hydrodynamic fluctuation. These non-linear variations were likely associated with tidal interaction, backwater effects, short-term rainfall-runoff variation, and rapidly changing coastal hydrological conditions.

For the hybrid solution, the results highlight the complementary roles of YOLOv10 and LSTM in the proposed framework. LSTM provides short-term predictive capability for proactive warnings, while YOLOv10 offers real-time visual validation of actual water levels, maintaining high reliability even as forecasting uncertainty increases. This hybrid design enhances system robustness by mitigating the limitations of individual data sources and models.

During the monitoring period, the system generated multiple flood alert events based on predefined thresholds and predictive outputs. These alerts were validated using staff gauge measurements and CCTV observations. The majority of alerts corresponded to genuine hydrological events, indicating a high true positive rate. False positives were minimal and primarily occurred during transient environmental fluctuations or sensor noise. The integration of YOLOv10 visual detection with LSTM forecasting significantly improved alert reliability by providing cross-modal verification.

Sensor readings were validated using staff-gauge observations and CCTV visual inspection. During heavy rainfall, measurement uncertainty may increase due to water-surface turbulence, splash effects, debris movement, and visibility degradation. Field observations indicated temporary deviations of approximately ±3–5 cm during periods of increased rainfall intensity. These effects were mitigated through signal filtering and cross-modal verification using YOLOv10 visual detection.

The system maintains operation under temporary network outages by leveraging edge computing. Local processing ensures continuous monitoring, while data buffering and optional local alert mechanisms (e.g., sirens or visual indicators) provide resilience in rural environments with unstable connectivity. In this work, we claim that the low, medium, and high warning levels are not fixed as universal values; rather, they are configurable site-specific thresholds derived from local staff-gauge readings, observed water-level ranges, and field calibration. At sites with available historical flood marks or topographic surveys, the thresholds should be recalibrated using the bankfull stage, cross-section geometry, and historical flood records.

However, the present field deployment captured multiple precipitation conditions, including light, moderate, and heavy rainfall events; however, repeated extreme rainfall events exceeding 100 mm within short durations were not consistently observed during the monitoring period. Since the experiments relied on real environmental conditions rather than controlled rainfall generation, validation under extreme hydrological scenarios remains a limitation.

Although the present deployment primarily employed 5G, future system extensions may integrate LPWAN technologies such as LoRaWAN to improve redundancy, energy efficiency, and long-range capabilities for rural deployments. Hybrid integration between LPWAN and 5G/6G infrastructures may further enhance scalability and operational resilience for large-scale AIoT disaster-monitoring systems.

## 8. Conclusions

This paper presented the development and field validation of an AIoT-based early flash-flood warning system for enhancing rural disaster resilience under practical environmental conditions. The proposed framework integrates multi-source IoT sensing, CCTV monitoring, edge computing, and hybrid AI models comprising YOLOv10-based visual flood detection and LSTM-based hydrological forecasting to provide both real-time situational awareness and short-term predictive warning capabilities.

Field deployments were conducted at the Ban Luang station in Chiang Mai and the Chumkho station in Chumphon under different hydrological and environmental conditions. The experimental results demonstrated that the YOLOv10 framework achieved high visual detection performance under daytime, nighttime, low-water, and high-water conditions. Although slight performance degradation was observed at night and under high-water conditions due to reduced illumination and environmental disturbances, the model maintained relatively high precision, recall, and mAP@0.5, making it suitable for practical flood monitoring applications.

The LSTM forecasting model achieved strong predictive performance at the Ban Luang station, with an R^2^ of 0.987, and moderate performance at the Chumkho station, with an R^2^ of 0.781. An additional error decomposition analysis demonstrated that the majority of prediction error at the Chumkho station occurred during transitional and flood-peak conditions, characterized by rapid hydrodynamic fluctuations, tidal interactions, and coastal backwater effects. Nevertheless, the proposed forecasting framework remained capable of tracking the overall temporal trend of water-level variation and providing operationally useful short-term warning information.

The proposed hybrid decision framework further integrated sensor measurements, YOLOv10-based visual detection, and LSTM-based forecasting outputs to generate multilevel warning decisions for real-time flood monitoring. Event-level validation during the 60-day deployment period demonstrated high operational reliability, with detection probabilities exceeding 0.92 and critical success indices exceeding 0.82 at both monitoring stations. In addition, the proposed framework achieved a low E2E warning latency of approximately 4–5 s by integrating edge computing, 5G communication, and LINE-based mobile notification dissemination.

The field deployment further demonstrated the practical feasibility of community-level warning dissemination through web-based dashboards and smartphone notification platforms. An operational resilience analysis showed that the proposed framework maintained system uptime exceeding 97% during the monitoring period despite temporary communication interruptions and unstable environmental conditions.

The results confirm that the proposed AIoT-driven hybrid sensing framework provides a scalable, low-cost, and reliable solution for the real-time early warning of flash floods in rural environments. Future work will focus on extending the deployment to additional hydrological regions, improving the adaptive AI learning capability, integrating hydrodynamic simulation models, and enhancing the communication resilience under severe disaster conditions.

## Figures and Tables

**Table 1:** Comparison of the existing flood early warning systems and the proposed AIoT framework.

Ref.	System Type	AI Model	Forecast Capability	Visual Detection	Warning Lead Time	FAR ^1^	E2E ^3^ Latency	Communication	Deployment Area
[8]	IoT-based	Fuzzy	No	No	Real-time	0.236	50–60 s	LoRa	Mountainous areas
[19]	IoT-based	No	No	No	Real-time	No	30 s	Wi-Fi	Agricultural areas
[24]	N/A	ANN, Fuzzy, and ANFIS	Yes	No	Real-time	No	N/A ^2^	N/A	River areas
[26]	IoT-based	LSTM	Yes	No	30 min	No	50 s	Wi-Fi	River areas
[27]	IoT-based	LSTM	Yes	No	30 min			Wi-Fi	Urban areas
[28]	IoT-based	DNN, LSTM	Yes	No	Real-time	No	N/A	N/A	Rural areas
[29]	AIoT-based	DeepLabv3	Yes	No	30 min	No	N/A	N/A	Urban areas
[30]	AIoT-based	Fuzzy + LSTM	Yes	No	30 min	0.215	30 s	Wi-Fi	Urban areas
[32]	N/A	CNN	Yes	CCTV	Real-time	No	20 ms	Optical	River areas
[37]	N/A	YOLOv8	No	Drone	Real-time	No	20 s	Wi-Fi	Urban areas
[38]	N/A	YOLOv8 + SVM	Yes	No	Real-time	No	12 s	Wi-Fi	Urban areas
DDPM ^4^	IoT-based	No	No	No	10–30 min	0.723	15 min	3G	Rural areas
This work	AIoT-based	YOLOv10 + LSTM	Yes	CCTV	Real-time	0.111	8–11 s	5G	Rural areas

^1^ False alarm ratio. ^2^ Not available. ^3^ End-to-End. ^4^ Department of Disaster Prevention and Mitigation.

**Table 2:** The comparative performance of YOLOv5, YOLOv7, YOLOv8, YOLOv9, and YOLOv10.

Model	Detector Type	Backbone	Neck	Label Assignment	Key Feature	Suitability for AIoT
YOLOv5 [42]	Anchor-based	CSPDarknet	PAN	OTA/default	Highly stable PyTorch 2.0 baseline	Good
YOLOv7 [43]	Anchor-based	E-ELAN	PAN	Coarse label assignment	Best training optimization	Very good
YOLOv8 [44]	Anchor-free	C2f	PAN/FPN	TAL	First fully anchor-free YOLO	Excellent
YOLOv9 [45]	Anchor-free	GELAN + PGI	FPN-PAN	TAL	Programmable gradient innovation	Excellent
YOLOv10	Anchor-free	C2fCIB + SCDown + PSA	Cross-scale Concat + SPPF	MSHA + PSA attention	Fastest hybrid efficiency	Outstanding for Edge-AI

**Table 3:** Specification of the CCTV image dataset for YOLOv10 visual flood detection.

Parameter	Description
▪ Total images	▪ 1200.
▪ Image source	▪ IP CCTV cameras at the Ban Luang and Chumkho stations.
▪ Lighting conditions	▪ Daylight, low-light, night infrared (IR).
▪ Weather conditions	▪ Clear, light rain, heavy rain, high humidity.
▪ Flow regimes	▪ Calm flow, turbulent flow, high discharge, sediment-laden floods.
▪ Annotation—bounding lines	▪ Staff gauge.
▪ Annotation—water level	▪ Water-level position on the gauge.
▪ Annotation—flood state	▪ Low level, high level.
▪ Image resolution	▪ 640 × 640 pixels.
▪ Data augmentation	▪ Random flips, brightness adjustment, noise injection, rain simulation.
▪ Dataset split	▪ 70% training/15% validation/15% testing.
▪ Maximum training epochs	▪ 2300.

**Table 4:** Specification of the multivariate hydrological time-series dataset for LSTM forecasting.

Parameter	Value	Description
▪ Sampling interval	1	▪ Temporal resolution of recorded sensor data.
▪ Look-back window (k)	24	▪ Uses the previous 24 h historical observations.
▪ Forecast horizon ($H_{sens}$)	1	▪ Predicts the water level 1 h ahead.
▪ Input features	5	▪ Water level, rainfall, temperature, humidity, and light intensity.
▪ Number of LSTM layers	2	▪ Stacked LSTM architecture.
▪ Hidden units (Layer 1)	128	▪ Captures long-term temporal dependencies.
▪ Hidden units (Layer 2)	64	▪ Refines temporal feature representation.
▪ Dense layer neurons	32	▪ Fully connected layer before output.
▪ Activation functio (σ)	ReLU	▪ Applied in a dense hidden layer.
▪ Optimizer	Adam	▪ Adaptive gradient optimization.
▪ Learning rate	0.001	▪ Initial learning rate.
▪ Batch size	32	▪ Number of samples per training iteration.
▪ Number of epochs	50	▪ Maximum training cycles.
▪ Number of datasets	2942	▪ Total dataset for both stations.
▪ Training samples	2043	▪ Split 70% ratios for training samples.
▪ Validation	438	▪ Split 15% ratios for validating.
▪ Testing	437	▪ Split 15% ratios for testing.
▪ Iteration	2300	▪ Number of training cycles.

**Table 5:** Water-level warning thresholds.

Station	Low	Medium	High
Ban Luang	$H_{sens}\left(t\right)$ < 0.5 m	0.5 ≤ $H_{sens}\left(t\right)$ < 0.8	$H_{vis}\left(t\right)$ ≥ 0.8
Chumkho	$H_{sens}\left(t\right)$ < 0.6 m	0.5 ≤ $H_{sens}\left(t\right)$ < 0.9	$H_{vis}\left(t\right)$ ≥ 0.9

**Table 6:** YOLOv10 performance score at the Ban Luang station under different environmental conditions.

Condition	Precision	Recall	mAP@0.5
Daytime-Low Water	1.000	0.980	1.000
Daytime-High Water	1.000	0.950	0.999
Nighttime-Low Water	0.962	0.931	0.957
Nighttime-High Water	0.948	0.915	0.941

**Table 7:** YOLOv10 performance score at the Chumkho station under different environmental conditions.

Condition	Precision	Recall	mAP@0.5
Daytime-Low Water	1.000	0.980	1.000
Daytime-High Water	1.000	0.950	0.999
Nighttime-Low Water	0.962	0.931	0.957
Nighttime-High Water	0.948	0.915	0.941

**Table 8:** Summary of the error decomposition analysis of the LSTM prediction performance at the Ban Luang and Chumkho stations under different hydrological conditions.

Station	Hydrological Condition	RMSE (m)	MAE (m)	R^2^ (m)
Ban Luang	Baseline condition	0.021	0.014	0.992
	Transitional rising-water period	0.039	0.026	0.989
	Flood-peak condition	0.062	0.043	0.978
	Overall performance	0.057	0.015	0.987
Chumkho	Baseline condition	0.028	0.021	0.912
	Transitional rising-water period	0.061	0.053	0.804
	Flood-peak condition	0.094	0.082	0.693
	Overall performance	0.051	0.050	0.781

**Table 9:** Event-level alert validation performance during 24 October–24 December 2025.

Station	Total Alerts	*TP*	*FP*	*FN*	*FAR*	*POD*	*CSI*
Ban Luang	18	16	2	1	0.111	0.941	0.842
Chumkho	27	24	3	2	0.111	0.923	0.828
Overall	45	40	5	3	0.111	0.930	0.833

**Table 10:** Representative fusion-score results for water-level alert events during 24 October–24 December 2025.

Station	Event Type	No. of Event	$S_{sens}$	$S_{LSTM}$	$S_{YOLO}$	$R_{t}$	$A_{t}$
Ban Luang	*TP*	16	0.589	0.662	0.792	0.677	Medium
Ban Luang	*FP*	2	0.342	0.461	0.712	0.503	Medium
Ban Luang	*FN*	1	0.781	0.623	0.382	0.593	Medium
Chumkho	*TP*	24	0.612	0.690	0.812	0.703	Medium
Chumkho	*FP*	3	0.363	0.492	0.691	0.513	Medium
Chumkho	*FN*	2	0.761	0.591	0.350	0.567	Medium
Overall	*TP*	40	0.602	0.683	0.802	0.693	Medium
Overall	*FP*	5	0.351	0.486	0.704	0.510	Medium
Overall	*FN*	3	0.770	0.605	0.363	0.577	Medium

**Table 11:** Component-wise E2E latency analysis of the proposed warning system during 24 October–24 December 2025.

Station	$T_{sens}$ (s)	$T_{edge}$ (s)	$T_{cloud}$ (s)	$T_{notify}$ (s)	$T_{Total}$ (s)
Ban Luang (Min)	0.42	0.88	0.74	0.69	3.08
Ban Luang (Max)	0.95	1.86	1.62	2.11	6.28
Ban Luang (Mean)	0.61	1.24	1.08	1.48	4.41
Chumkho (Min)	0.38	0.79	0.61	0.73	2.51
Chumkho (Max)	1.21	2.44	2.15	3.09	8.89
Chumkho (Mean)	0.68	1.36	1.21	1.48	4.73

**Table 12:** System resilience and operational reliability during the 60-day deployment period.

Parameter	Ban Luang Station	Chumkho Station
▪ Monitoring duration	60 days	60 days
▪ System uptime	98.9%	97.8%
▪ Communication outage events	3	5
▪ Longest outage duration	12 min	21 min
▪ Mean outage duration	5.3 min	8.7 min
▪ Data loss during outage	None	None
▪ Edge buffering	Enabled	Enabled
▪ Automatic cloud resynchronization	Enabled	Enabled
▪ Backup battery operation	Supported	Supported

## Data Availability

The data presented in this study are available from the corresponding author upon request.

## Abbreviations

The following abbreviations are used in this manuscript:

AIoT	Artificial Intelligence of Things
IoT	Internet of Things
LoRaWAN	Long-range wide area network
YOLO	You Only Look Once
LSTM	Long short-term memory
CCTV	Closed-circuit television
NVR	Network video recorder
EFFWS	Early flash-flood warning system
SAEO	Speed–accuracy efficiency optimization
RNN	Recurrent neural network
PAN	Path aggregation network
DNN	Deep neural network
CNN	Convolution neural network
ANN	Artificial neural network
SVM	Support vector machine
ANFIS	Adaptive neuro-fuzzy inference system.
WNN	Wavelet neural networks
E-ELAN	Extended efficient layer aggregation network
FPN	Feature pyramid network
GELAN	Generalized efficient layer aggregation network
PGI	Programmable gradient information
TAL	Task alignment learning
OTA	Optimal transport assignment
mAP	Mean average precision
RMSE	Root mean square error
MAE	Mean absolute error
IR	Infrared
LAN	Local area network
